# *A*ntibiotics and *S*urgical *S*ite Infection in *E*xpander-Based Breast *R*econstruction *T*rial (ASSERT)

**DOI:** 10.1245/s10434-025-18472-6

**Published:** 2025-10-14

**Authors:** Surinder Kaur, Brian Gastman, Kristen P. Broderick, Adeyiza O. Momoh, Brett T. Phillips, Graham Schwarz, Summer E. Hanson, Geoffrey E. Hespe, Carisa M. Cooney, Katie Sommers, Cheng-Shiun Leu, Christine H. Rohde

**Affiliations:** 1 The Plastic Surgery Foundation, Arlington Heights, IL USA; 2 Cleveland Clinic, Cleveland, OH USA; 3 Iovance Biotherapeutics, Philadelphia, PA USA; 4 Johns Hopkins University School of Medicine, Baltimore, MD USA; 5 University of Michigan Medical Center, Ann Arbor, MI USA; 6 Duke University Medical Center, Durham, NC USA; 7 The University of Chicago Medical Center, Chicago, IL USA; 8 Columbia University Mailman School of Public Health, New York, NY USA; 9 Columbia University Irving Medical Center, New York, NY USA

## Abstract

**Background:**

The use of prophylactic antibiotics following postmastectomy tissue expander breast reconstruction (TE-BR) varies widely. The Centers for Disease Control and Prevention (CDC) recommends a single preoperative antibiotic dose for clean and clean-contaminated procedures. This multi-institutional, prospective, randomized controlled trial (RCT) examined whether the CDC-recommended single preoperative dose (SPD) of antibiotics is not inferior to an additional week of postoperative (WPO) prophylactic antibiotics in preventing surgical site infection (SSI) in immediate TE-BR following mastectomy.

**Methods:**

Women aged ≥ 18 years undergoing immediate TE-BR were randomized to SPD or WPO groups. The primary outcome was SSI by CDC guidelines within 30 days of surgery. The study used a noninferiority trial design to examine whether the test product (single preoperative dose (SPD)) was not worse than the comparator (1 week of postoperative (WPO) prophylactic antibiotics) by more than a set noninferiority margin of 6%.

**Results:**

In total, five participating centers screened 499 women; 235 were enrolled. A total of 102 patients were randomized to the SPD arm and 112 to the WPO arm, with 21 patients withdrawn. The SSI rate in the SPD arm was 17% as compared with 11% in WPO arm, which is within the noninferiority margin set for this study but not significantly noninferior (*p *= 0.496). The rate of unplanned TE removal for infection, hospitalization rate, and return to OR rate within 30 days of surgery were comparable between the two groups.

**Conclusions:**

This multi-institutional RCT did not definitively demonstrate that a single preoperative dose of antibiotics is not inferior to a 7-day postoperative antibiotic regimen in preventing SSI in immediate TE-BR; there was also no evidence to support that the 7-day regimen was significantly better. As this represents one of the largest multi-institutional study of its kind, these results have practice-management considerations.

**Supplementary Information:**

The online version contains supplementary material available at 10.1245/s10434-025-18472-6.

Surgical site infection (SSI) is likely one of the most common complications in all of surgery. SSIs can impact patients’ mental and physical outcomes and contribute to rising healthcare costs.^1,2^ Plastic and reconstructive surgery frequently employs implanted alloplastic materials, which has a higher risk of SSI compared with non-implant-based surgeries. In tissue expander breast reconstruction (TE-BR), the reduced vascularity of the soft tissue envelope around the implant places these devices at even higher risk of infection and exposure. Infection and loss of an implant contribute to patient distress and often necessitate the use of secondary surgical options and can delay definitive reconstruction and adjuvant cancer therapies.^3^ Because of these concerns, plastic surgeons have historically used a course of postoperative prophylactic antibiotics after TE-BR. Based on the literature and from self-reporting from the American Society of Plastic Surgery (ASPS) membership, there is a wide range of prophylactic antibiotic use to prevent SSI from as little as one preoperative dose to many weeks of therapy; 1 week of postoperative antibiotics is the most common.^4–6^ The CDC created a national action plan to reduce unnecessary prophylactic antibiotics for clean noncontaminated cases by advocating the use of a single preoperative dose of antibiotics.^7^ This revised guideline states that patients undergoing clean and clean-contaminated procedures do not need to receive any doses of antibiotic after incision closure, even if a drain is present. For all patients, intraoperative redosing is needed to ensure adequate serum and tissue concentrations of the antimicrobial if the duration of the procedure exceeds two half-lives of the drug. This plan was implemented to reduce the negative consequences of antibiotics overuse, which can lead to the development of bacterial resistance and adverse events.^7–9^ Currently, there is mounting pressure on the plastic and reconstructive surgery community to adopt a data-driven rationale for SSI prophylaxis.^8,9^

Implant-based surgery has unique risks for infection, and SSI in this setting can lead to loss of or delay in reconstruction, increase in reconstruction costs, delay in start of adjuvant therapy, and poor patient satisfaction.^2,3,10^ It is thus intuitive that some form of antimicrobial prophylaxis is required. This need must be balanced by well-documented risks of overtreatment with antimicrobials.^11^ Fields such as orthopedic surgery^12^, where implants are used, and colorectal surgery, where contamination can occur, have performed randomized controlled trial (RCT) analyses, which reveal that a single dose of prophylactic antibiotics is noninferior to additional postoperative doses.^13,14^ While there is robust literature on this topic, plastic surgery procedures such as implant-based breast reconstruction have several unique features that make findings from other surgical specialties difficult to translate. Some of these differences include two distinct surgical teams operating in the same area, with potentially different standards of tissue handling and infection control.^15^ The desire for an esthetic and uncomplicated breast reconstruction may conflict with the breast surgeon’s goal of a complete mastectomy with minimal risk of recurrence.^15,16^ Another potential issue facing plastic surgeons is the loss of the tissue envelope covering the implant with variable vascularity to the skin flaps which can change even more with tissue expansion. Mastectomy skin flap thickness and surgeon/patient preference play important roles in the operative plan.^16,17^

Patient and treatment factors carry the highest risk of SSI in breast reconstruction.^18^ Mastectomy weight and BMI have been shown to be positive predictors of complications after immediate tissue expander (TE) reconstruction.^19^ Significant preoperative selection factors have been identified to contribute to the elevated risk of infection, such as older age, higher BMI, diabetes, and smoking status^20^. Significant intraoperative and postoperative contributing factors include greater mastectomy weight, larger TEs and intraoperative fill volume, and longer drain duration.^20,21^ Direct-to-implant (DTI) surgery is suitable for those undergoing nipple-sparing mastectomies or those who prefer a single surgery approach. Although it has similar complication rates as TE-BR, it may be associated with higher postoperative wounds and capsular contracture.^22,23^

Definitive studies to determine an optimal therapeutic strategy to prevent SSI in implant-based plastic surgery procedures are lacking.^5,6,9,24–29^ Only a few studies exist that attempt to determine the optimal prophylactic antibiotic regimen for TE-BR.^9,27–29^ Most published prospective studies have occurred in one or two institutions and/or had relatively small sample sizes.^26,27^ However, consistent with CDC recommendations, there is mounting evidence that a single preoperative dose for alloplastic breast reconstruction may be adequate.^26,30–33^ The goal of this multi-institutional, prospective, RCT is to examine whether the CDC-recommended single preoperative dose (SPD) of antibiotics is not inferior to 1 week of postoperative (WPO) prophylactic antibiotics in preventing SSI in immediate TE-BR following mastectomy. In embarking on this SSI study, we sought to perform a potentially practice-changing multi-institutional RCT leveraging the resources of The Plastic Surgery Foundation (PSF) and geographically diverse academic medical centers.

## Methods

### Study Design

This study is a prospective, multi-institutional, noninferiority RCT comparing two prophylactic antibiotic dose regimens in patients receiving immediate TE-BR. We aim to examine whether the test product (SPD) is not worse than the comparator (WPO) by a noninferiority margin (*δ *= 6% for this study) (see ASSERT Study Protocol, Supplementary Digital Content 1). The SPD group received a single preoperative dose of intravenous (IV) antibiotic only (CDC recommendation) with additional intraoperative doses as recommended based on operative time. The WPO group received a single preoperative dose of IV antibiotic with additional intraoperative doses as recommended based on operative time, plus 7 days of oral postoperative antibiotics (cephalexin: 500 mg by mouth (PO) four times daily (qid) or every 6 h (q6h); cefadroxil: 500 mg PO twice daily (bid); or clindamycin: 300 mg PO qid or q6h). In cases of allergies to these antibiotics, antibiotics of physician choice (such as trimethoprim/sulfamethoxazole or doxycycline with standard dosing) were used. These antibiotics are effective against *Staphylococcus aureus*, *Streptococcus*, coagulase-negative staphylococci, and other skin flora that are most commonly associated with SSIs in mastectomy and implant-based breast reconstruction.

### Inclusion/Exclusion Criteria

Adult (≥ 18 years) women undergoing unilateral or bilateral mastectomy (including breast cancer, any stage, or prophylactic) and immediate TE-BR (including submuscular with or without acellular dermal matrix (ADM) or prepectoral placement with or without ADM) were approached for enrollment. Since the receipt of unilateral or bilateral mastectomy with reconstruction is the inclusion criteria, only female patients were eligible for this study. Patients were excluded if they were not undergoing mastectomy or were undergoing direct-to-implant reconstruction, delayed reconstruction, or autologous reconstruction; had a history of chest wall radiation; or had a history of previous breast reconstruction on the side of expander placement. Smoking status was not an exclusion criterion and was self-reported by patients.

### Study Procedures

Patients were enrolled at five reconstructive centers across the country: Cleveland Clinic, Duke University, Johns Hopkins University School of Medicine, University of Chicago, and University of Michigan. Sites were chosen based on volume of procedures, number of potential participating surgeons, and willingness to randomize patients. Each site had at least one institutional investigator responsible for local compliance. This study was approved by the Western Institutional Review Board as the central institutional review board (IRB) and registered at clinicaltrials.gov (NCT-04631185). The patients were enrolled from 1 May 2021 to 31 May 2023.

Eligible patients who agreed to participate signed the study informed consent form and were randomized to either the SPD or WPO group using permuted block randomization stratified by site to ensure balance on treatment allocation within each site. Based on the design of this study, neither the surgeon nor the patient was blinded to allocation.

Intravenous cefazolin (2 g for patients up to 120 kg) was prescribed to all patients before surgery. In cases of an allergy to cefazolin, intravenous clindamycin was prescribed, and in the rare cases where neither could be used, an appropriate antibiotic was chosen by the surgeon. All patients received a single (weight-based) dose of antibiotic within 60 min prior to incision; any intraoperative doses of antibiotics were administered according to current recommendations based on the antibiotic’s dosing and operative time. Skin preparation was performed using chlorhexidine-based sterilization. Pocket irrigation was according to surgeons’ standard practice (triple antibiotics, bacitracin, betadine, saline, or other). Other operative details were according to the surgeons’ standard procedure. Race and ethnicity information was not collected in this study.

### Study Visits

All patients had their first follow-up by 2 weeks postoperatively and a second visit within 30 (± 7) days postoperatively. At the first postoperative visit, patients assigned to receive 1 week of postoperative antibiotics were asked to report which doses they took. The patients were followed until TE removal/exchange or the study end, whichever was earlier.

### Sample Size

Sample size calculations were based on estimates from previous studies and considered 12% versus 18% SSI rate for the postoperative antibiotic (reference group) and CDC recommendations (under the null hypothesis of inferiority), respectively. We planned a noninferiority design with a clinically meaningful noninferiority margin of *δ *= 6% between the group proportions. Estimates were computed using one-sided *z*-test (unpooled), with the significance level targeted at 0.025. To conclude noninferiority of the CDC recommended single dose compared with the postoperative antibiotic course at 80% power, a total of 922 patients would be needed (461 per arm). Accounting for dropouts, the plan was to recruit 500 patients per group (1000 total). The trial was stopped on 31 May 2023 with 235 total patients enrolled because the study’s funding ran out.

### Data Collection and Data Analysis

Each site collected data using a standardized case report form (CRF) and entered into a secure and Health Insurance Portability and Accountability Act (HIPAA)-compliant central study database set up in REDCap^34^ by the Plastic Surgery Foundation (PSF). The database was accessible to authorized users via secure web access, and site personnel only had access to their site’s data. The primary endpoint was SSI as defined by CDC criteria^7,35^ within 30 days after the index procedure (see SSI Definition by CDC Criteria, Supplementary Digital Content 2); single per-patient-SSI was defined as a unilateral or bilateral infection of the surgical site. The secondary endpoints were (1) SSI beyond 30 days after index procedure up to time of tissue expander exchange (for an implant or flap), (2) number of hospitalizations, (3) tissue expander or implant loss, (4) antibiotic and wound culture speciation and antibiotic sensitivities in SSI/deep infections. An SSI designation was based on CDC definition and site investigator assessment. Despite the CDC guidelines on SSI definition, the clinical diagnosis of SSI occasionally may not be clear-cut. Any SSI determination that was not clear-cut was reviewed by all investigators to make sure that a majority agreed with that diagnosis. Additionally, mastectomy skin flap necrosis can sometimes be the driver for expander removal rather than an infection. These observations were accurately recorded in the CRF and helped in determining that an outcome was related to an SSI. Overall, investigator judgment of appropriate clinical care determined the management of postoperative signs and symptoms. Antibiotic-related adverse events, e.g., rash, gastrointestinal symptoms, colitis, yeast infections, anaphylaxis, etc., were also assessed and documented. Descriptive statistics were generated and compared for patient demographic characteristics as well as operation details by treatment assignment (SPD versus WPO) using the *t*-test for continuous variables and chi-squared test for categorical variables. Since several demographic variables had cell frequencies of five or fewer, Fisher’s exact test was also performed for those dichotomous variables. Following intent-to-treat (ITT) principle, we used a *z*-test to test the null hypothesis that *P*_WPO_–*P*_SPD_ ≤ − 0.06 for the infection-related endpoints (both primary and secondary) where *P*_WPO_ and *P*_SPD_ are the true probability of infection for WPO and SPD groups, respectively. As the majority of study patients followed the protocol completely (five patients did not follow the protocol, one in SPD arm received antibiotics and four in WPO arm did not get antibiotics), the results for per-protocol (PP) analysis were very similar to those from ITT analysis. Therefore, only ITT analysis results are reported in this paper. While descriptive statistics were also reported for other non-infection-related endpoints, we did not conduct statistical comparisons between the two groups, as the margin of inferiority was not prespecified in the protocol for those variables. Analysis of primary and secondary outcomes was also conducted by ADM use (yes versus no) and by TE placement (submuscular versus prepectoral). We employed logistic regression analysis to examine potential factors that may associate with developing SSI within 180 days of surgery. Finally, we generated Kaplan–Meier curves to provide a graphical representation of the time to SSI within 180 days by treatment group and compared the two survival curves via the log-rank test. We declared a finding as statistically significant if its corresponding *p*-value was no greater than 0.05. Analysis was conducted using SAS Version 9.4.

## Results

**Fig. 1:**
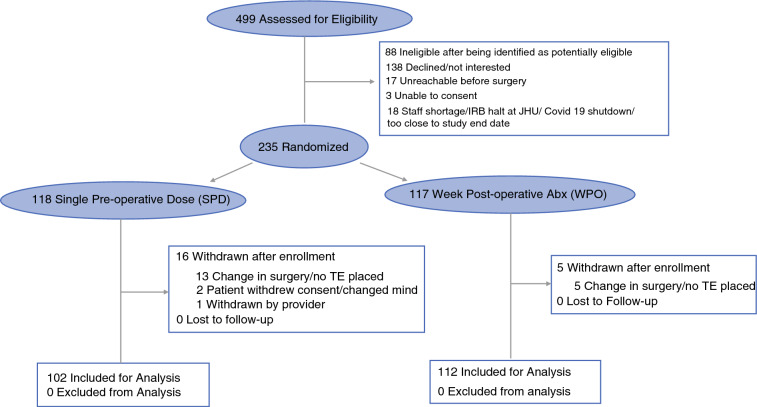
Consort diagram

Between May 2021 and May 2023, 499 eligible patients at five study centers were approached for enrollment; 235 were enrolled (Fig. 1). Of these, 118 patients were randomized to the SPD arm and 117 to the WPO arm. In total, 21 patients were withdrawn after enrollment: 18 had a change in surgery and no TE placement, two patients withdrew consent, and one was withdrawn by the provider for needing additional intraoperative antibiotics. Data from 102 patients randomized to the SPD group and 112 to the WPO group were used for analysis.

Demographic characteristics were similar between both groups (Table 1). The mean patient age at time of operation was 49 years; 27% of SPD patients and 24% of WPO patients had a body mass index (BMI) > 30. The distribution of other risk factors such as smoking status, diabetes mellitus, hypertension, cardiac disease, and other comorbidities in the two groups were also similar.

**Table 1:** Patient demographics

Characteristics	SPD *n *= 102	WPO *n *= 112	*p*-Value (chi-squared test)	*p*-Value (Fisher exact test)
Patients, *n*	102 (100)	112 (100)	n/a	n/a
Age (years) mean (SD), range	48.6 (10.5), 28–71	49.1 (11.2), 22–78	0.751	n/a
Obesity (BMI > 30 kg/m^2^), *n* (%)	27 (26.5)	27 (24.1)	0.691	0.753
Smoking status			0.077	n/a
Nonsmoker, *n* (%)	73 (71.6)	81 (72.3)		
Past smoker, *n* (%)	29 (28.4)	26 (23.2)		
Current smoker, *n* (%)	0 (0)	5 (4.5)		
Comorbidities				
Comorbidity present, yes *n* (%)	41 (40.2)	49 (43.8)	0.599	0.678
Hypertension, *n* (%)	14 (13.7)	20 (17.9)	0.409	0.999
Diabetes mellitus, *n* (%)	4 (3.9)	6 (5.4)	0.619	0.751
Cardiac disease, *n* (%)	2 (2.0)	3 (2.7)	0.728	0.457
Renal disease, *n* (%)	2 (2.0)	1 (0.9)	0.507	0.606
Other comorbidities, *n* (%)	30 (29.4)	33 (29.5)	0.993	0.999
Antibiotic allergy				
Antibiotic allergy, *n* (%) yes	26 (25.5)	35 (31.5)	0.351	0.367
Neoadjuvant chemotherapy			0.312	n/a
Neoadjuvant chemotherapy, yes	21 (20.6)	28 (25.0)		
Neoadjuvant chemotherapy, no	76 (74.5)	76 (69.6)		
Neoadjuvant chemotherapy, unknown	5 (4.9)	6 (5.4)		

*n/a* not available

Operative and treatment characteristics were comparable between both groups (Tables A–C, Supplementary Digital Content 3). The percentages of mastectomy types (skin-sparing, nipple-sparing, and other) and drain usage (number and duration of drain placement) in both groups were similar (Tables A and C, Supplementary Digital Content 3). In total, 70% of patients had bilateral mastectomies, and 50% of the patients in both groups had prophylactic mastectomies. Cefazolin was used as intraoperative antibiotic in 88% of the patients, clindamycin in 10%, and other antibiotics for 2%. ADM was used in 75% of the operations; 90% of the TE placements were prepectoral.

Complications were reported for 41% (*n *= 42) of SPD patients and 42% (*n *= 47) of WPO patients. Surgical site infection (including cellulitis and soft tissue infection) and seroma were some of the most frequently reported complications (Table 2). Overall return to operating room (OR) was higher in the WPO group (16%, *n *= 18) as compared with the SPD group (11.8%, *n *= 12). A total of 17% (*n *= 17) of the SPD group patients and 11% (*n *= 12) of the WPO group patients developed SSI within 30 days of surgery (Table 3); this difference was within the noninferiority margin set for this study but not statistically significant (*p *= 0.496). The patients were also followed for development of SSI beyond 30 days. Follow-up visit data was collected virtually in a few cases where there were no postoperative complications. The number of virtual visits was extremely small, and the reported incidence of SSI is not anticipated to be affected by that. In total, 24% (*n *= 24) of the SPD group patients and 18% (*n *= 20) of the WPO group patients developed infection within 180 days from surgery, which was again within the noninferiority margin set for the study but not significant (*p *= 0.476). We also tested the null hypothesis of no difference for each of the above outcomes; no significant differences were found. Of those who developed infections, 75% (*n *= 18) of SPD patients and 75% (*n *= 15) of WPO patients were treated with oral antibiotics; 54% (*n *= 13) of SPD patients and 45% (*n *= 9) of WPO patients also received IV antibiotics. The oral and IV antibiotics were according to physician’s standard practice, and the duration of antibiotics varied on the basis of the physician assessment of the patient and the severity of infection. The most commonly used antibiotics for treating infections were trimethoprim/sulfamethoxazole, ciprofloxacin, and vancomycin.

**Table 2:** Postoperative complications

Complications	SPD *n *= 102	WPO *n *= 112
Complications present: yes *n* (%)	42 (41.2)	47 (42)
Complication types		
Cellulitis, *n* (%)	19 (18.6)	10 (8.9)
Infection, *n* (%)	10 (9.8)	11 (10)
Seroma, *n* (%)	17 (16.7)	23 (20.5)
Return to OR, *n* (%)	12 (11.8)	18 (16.1)
Mastectomy skin flap necrosis, *n* (%)	7 (6.9)	5 (4.5)
Skin necrosis, *n* (%)	7 (6.9)	7 (6.3)
Dehiscence, *n* (%)	5 (4.9)	6 (5.4)
Hematoma, *n* (%)	4 (3.9)	8 (7.1)
Tissue expander removal, not due to infection, *n* (%)	4 (3.9)	3 (2.7)
Wound revision, *n* (%)	0 (0.0)	4 (3.6)
Surgical treatment, *n*		
Tissue expander removal, *n* (%)	21 (20.6)	20 (17.9)
Wound revision in OR, *n* (%)	8 (7.8)	12 (10.7)
Incision or drainage in clinic, *n* (%)	5 (4.9)	6 (5.4)
Wound revision in clinic, *n* (%)	4 (3.9)	0 (0.0)
Implant removal, *n* (%)	0 (0)	2 (1.8)

**Table 3:** Primary and secondary endpoints

Endpoints	Intervention (SPD *N *= 102)	Control (WPO *N *= 112)	*p*-Value^*^
Primary endpoint			
SSI: infection within 30 days,* n* (%)	17 (17%)	12 (11%)	0.496
Secondary endpoints			
Infection within 90 days, *n* (%)	23 (23%)	18 (16%)	0.535
Infection within 180 days, *n* (%)	24 (24%)	20 (18%)	0.476
TE removal for infection within 30 days	6 (6%)	2 (2%)	0.232
Any TE Removal for infection	18 (18%)	13 (12%)	0.503
Return to OR within 30 days, *n* (%)	11 (11%)	11 (10%)	
Hospitalization within 30 days, *n* (%)	9 (9%)	7 (6%)	
Hospitalization between 31 and 90 days, *n* (%)	6 (6%)	8 (7%)	

Treatment if yes to infection (*N *= 44)	Intervention (SPD *N *= 24)	Control (WPO *N *= 20)
Intravenous antibiotic, *n* (%)	13 (54%)	9 (45%)
Oral antibiotic, *n* (%)	18 (75%)	15 (75%)
Surgical intervention, *n* (%)	16 (67%)	14 (70%)
Close observation only, *n* (%)	0 (0%)	0 (0%)

^*^p-value was corresponding to test the null hypothesis that *P*_WPO_ − *P*_SPD_ ≤ −0.06 for the infection-related endpoints, where *P*_WPO_ and *P*_SPD_ are the true probability of infection for WPO and SPD group respectively. *p*-values for the remaining analyses were not provided as the margin of inferiority was not prespecified

The rate of TE removal for infection within 30 days of surgery was 6% (*n *= 6) in the SPD group compared with 2% (*n *= 2) in the WPO group (*p *= 0.232) (Table 3). Overall TE removal was 18% (*n *= 18) in the SPD group and 12% (*n *= 13) in the WPO group (*p *= 0.503). Hospitalization rate (9% in SPD versus 6% in WPO) and return to OR rate (11% in SPD versus 10% in WPO group) within 30 days of surgery were comparable within the two groups. *p*-values for these analyses were not calculated as the margin of noninferiority was not prespecified in the protocol for secondary outcomes.

**Fig. 2:**
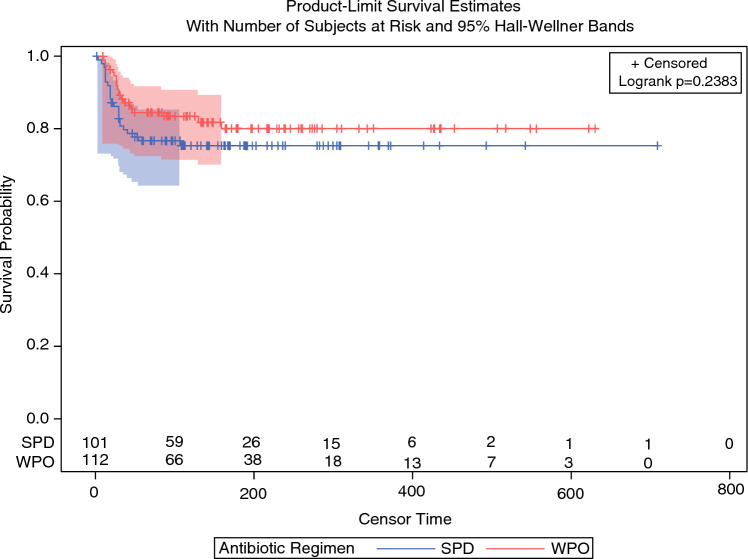
Kaplan–Meier (KM) curves for infection-free survival

Figure 2 shows the infection-free survival in SPD versus WPO groups. The number of patients at risk at day 100 is comparable in SPD (58.4%, *n *= 59) and WPO (58.9%, *n *= 66) groups. We found no statistically significant difference between the two survival curves (*p *= 0.238).

**Fig. 3:**
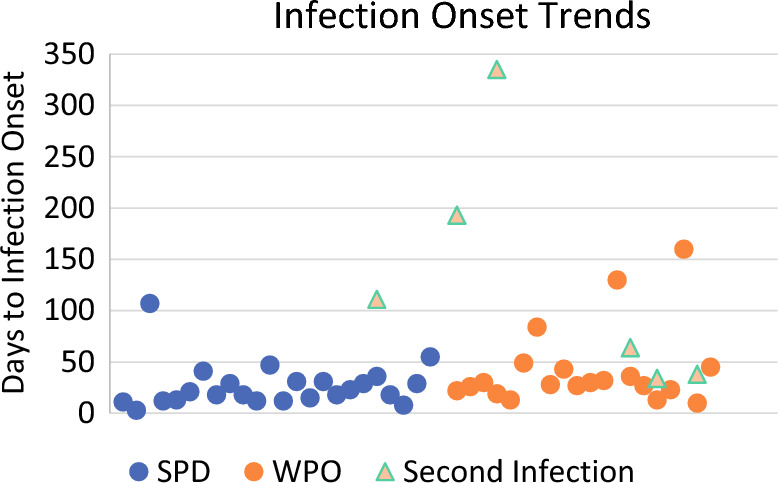
Infection onset trends

Infections in the SPD group were seen earlier than the WPO group. The median time to SSI onset was 21 days (*n *= 24) in the SPD group and 29 days (*n *= 20) in the WPO group. The WPO group also showed a higher number of repeat infections (4.5%, (*n *= 5)) compared with the SPD group [1%, (*n *= 1)] (Fig. 3). Wound culture results from five of the six patients who had repeat infections in WPO group were positive for one or more of the following: *Pseudomonas,* methicillin-resistant *Staph. aureus,* methicillin-sensitive *Staph. aureus*, or *Serratia marcescens*. The one patient in SPD group that had a repeat infection was wound-culture negative.

In total, 28 SPD patients received oral antibiotics owing to SSI or suspected SSI; nine SPD patients (9%) received both oral and IV antibiotics for treating SSI. A total of 16.7% of SPD patients as compared with 14.3% of WPO patients were wound-culture positive. Only three patients in WPO group had antibiotic-related complications (rash and nausea/vomiting) (Table 4).

**Table 4:** Antibiotic-related complications

Antibiotic related complications	SPD *n *= 102	WPO *n *= 112
Antibiotic related complications yes, *n* (%)	0 (0.0)	3 (2.7)
Antibiotic related complications no, *n* (%)	102 (100)	108 (96.4)
Antibiotic related complications unknown, *n* (%)	0 (0.0)	0 (0.0)
Complication types		
Rash, *n* (%)	0 (0.0)	2 (1.7)
Nausea/vomiting, *n* (%)	0 (0.0)	1 (0.9)
Diarrhea, *n* (%)	0 (0.0)	0 (0.0)
* C. difficle*, *n* (%)	0 (0.0)	0 (0.0)
Urinary tract infection, *n* (%)	0 (0.0)	0 (0.0)
Gastrointestinal upset, *n* (%)	0 (0.0)	0 (0.0)
Secondary infection, *n* (%)	0 (0.0)	0 (0.0)
Fungal infection, *n* (%)	0 (0.0)	0 (0.0)
Yeast infection, *n* (%)	0 (0.0)	0 (0.0)
Other, *n* (%)	0 (0.0)	0 (0.0)
Wound-culture positive, *n* (%)	17 (16.7)	16 (14.3)
Pseudomonas, *n* (%)	5 (4.9)	3 (2.7)
Methicillin-susceptible *Staphylococcus aureus* (MSSA), *n* (%)	4 (3.9)	4 (3.6)
Methicillin-resistant *Staphylococcus aureus* (MRSA), *n* (%)	1 (1)	1 (0.9)
S. epidermidis, *n* (%)	2 (2)	0 (0)
Enterococcus, *n* (%)	1 (1)	0 (0.0)
Enterobacter, *n* (%)	0 (0)	3 (2.7)
Other, *n* (%)	6 (5.9)	9 (8)

Increasing age showed some association with developing SSI within 180 days of surgery, but the results were not significant (*p *= 0.315). Increasing BMI (*p *= 0.048) and increasing mastectomy weight (*p *= 0.033) showed a significant association with developing an SSI within 180 days of surgery. Increasing TE size also increased the odds of getting an SSI with 180 days of surgery with a *p*-value trending toward significance (*p *= 0.105) (Table 5). The subgroup analysis with and without ADM use did not show higher SSI rates in the ADM group, though the *p*-values were not significant (Table, Supplementary Digital Content 4). The subgroup analysis for the comparison of SSI rates in prepectoral versus submuscular placement of TE also did not yield significant results owing to the small sample size for the submuscular group (Table, Supplementary Digital Content 5). Antibiotic types used to treat infections in the two groups are shown in Table, Supplementary Digital Content 6.

**Table 5:** Association of age, BMI, mastectomy weight and TE size with SSI within 180 days

Parameter	Exp(B)	95% Wald Confidence Interval for Exp(B)		Sig.
		Lower	Upper	
Age (years)	1.015	0.986	1.044	0.315
BMI (kg/m^2^)	1.059	1.001	1.120	0.048
Mean mastectomy weight (g)	1.001	1.000	1.002	0.033
Mean tissue expander size (mL)	1.002	1.000	1.005	0.105

Type of skin preparation, pocket irrigation, drain dressing, ADM use, TE placement (submuscular versus prepectoral), breast cancer status, and duration of surgery did not yield any significant association with development of SSI within 180 days (Tables, Supplementary Digital Content 7–9).

## Discussion

In this prospective, multi-institutional, noninferiority RCT comparing two prophylactic antibiotic dose regimens in patients receiving immediate TE-BR, we found that the difference in 30-day SSI rates for patients receiving a single preoperative dose (17%) did not exceed the noninferiority margin (6%) of those patients who received 1 week of postoperative oral antibiotics (11%).

Plastic surgery literature on optimal antibiotic prophylaxis is very variable due to mostly retrospective reporting, heterogeneity of study design and follow-up, as well as lack of randomization.^36,37^ One retrospective study found that the implementation of SSI improvement protocols and the resulting 24-h limited postoperative antibiotic course was associated with a significant increase in tissue expander reconstruction infections, compared with historical controls of giving antibiotics until drains were removed.^5^ In a retrospective comparison of a cohort of 134 patients who received antibiotics until drains were removed, versus a second cohort of 116 patients who received only a single preoperative dose of antibiotics,^5^ the preoperative antibiotic only group had an overall infection rate of 34.3% compared with the control group with 18.1% (*p *= 0.004)^5^.

In contrast to these studies, a retrospective market database study of over 7000 patients found no difference in SSIs or prosthesis explantation between patients who took postoperative antibiotics and those who did not.^25^ A systematic review of 81 studies suggested that there was no benefit to antibiotics beyond 24 h.^38^ Additionally, a noninferiority RCT in 112 patients undergoing TE-BR with ADM found no significant difference between a 24-h or prolonged course of antibiotics^26^. This study used a clinically wide noninferiority margin of 12% ± 10%, implying that an infection rate that differed from 2 to 22% between the two groups would still be considered statistically noninferior.^26^ This wide margin enabled a smaller sample size but is arguably too wide for clinical significance. Interestingly, patients in the less-than-24-h group were found in general to have earlier infections that were more likely to resolve with oral antibiotics. The longer-antibiotic-duration group had more patients with later infections that more often resulted in expander loss; however, these were trends only, without statistical significance. Despite the findings of these studies, the risks associated with antibiotic overuse, and the lack of literature supporting longer antibiotic courses, extended antibiotic regimens continue to be commonly employed by plastic surgeons.^39,40^ A multi-institutional randomized RCT is an opportunity for practice-changing results.

Much of the limitations of prior studies directly relate to the well-documented level of variability amongst plastic surgeons performing implant-based breast reconstruction, including the use of ADMs, differences in mastectomy skin flap thickness and vascularity, pre- or sub-pectoral placement of the expanders, and antibiotic use; in addition, there is variability in the definition of the “implant” (expander, permanent implant, etc.).^40–44^ To address the variability observed in these reports we employed a large RCT to minimize bias and enable better control of variables, especially by performing closer and longer follow-up. Direct-to-implant operations are significantly different than expander operations and have different complication profiles, for example, a higher incidence of capsular contracture and wounds.^23,24^ DTI was therefore an exclusion criteria, mainly to reduce variability in a study already containing variability based on location of expander (subpectoral or prepectoral) and ADM use.

In our study, we screened almost 500 patients but enrolled about half of that number (although this represents over 360 breasts studied), which illustrates the effort and difficulties with RCTs, especially in a field that rarely utilizes them. The RCT by Gahm et al.^33^ also did not achieve the planned sample size. It took them 5 years across seven hospitals to screen 711 patients undergoing DTI and TE-BR. We screened 499 patients undergoing TE-BR in effectively 1 year, across five hospitals, with delays and intermittent halts due to the coronavirus disease 2019 (COVID-19) pandemic. Our study also differed in using only TE-BR and not DTI, since those operations and risk profiles differ significantly.^23,24^ Their primary endpoint was TE or implant removal, not SSI, and the expanders were primarily subpectoral with no ADM; therefore, we would expect lower rates of SSI/implant removal. However, their rates of oral antibiotics for suspected SSI were 30.4% (single dose) and 24.1% (multi-dose),^33^ which were higher than our study’s 27% (SPD) and 21% (WPO). Still, neither study demonstrated a statistically significant difference between single dose or multidose antibiotic regimens.

While mainly a numerical finding (not statistically significant), the numbers of infection and resultant additional events seem to occur later in the WPO group, a finding also noted in the smaller RCT mentioned previously.^26^ This finding suggests that longer courses of antibiotics may only temporarily suppress potentially virulent bacteria that then can manifest later as an SSI with potentially more resistant or aggressive organisms; this may explain why the WPO patients had more repeat infections. Prolonged use of antibiotics does not have effect on *Pseudomonas,* methicillin-resistant *Staphylococcus aureus*, or *Serratia marcescens*.^27^ Culture and sensitivity assays should help in making appropriate changes in antibiotic management in such cases.^27^ It is worth noting that delayed SSI in the WPO group could be compounded by patients not having the same frequency of in-office follow-up at later postoperative time points. This is concerning, as delay in diagnosis for patients whose SSI occurs beyond the immediate postoperative period could lead to the need for hospitalization and the increase in implant loss.

Plastic surgery is a rapidly changing field, and from the initial conception to the actual conduct of the study, the rate of prepectoral placement of expanders quickly overtook the rate of total subpectoral or dual plane (subpectoral with ADM). Theoretically, the submuscular placement of implants may mitigate wound complications related to mastectomy skin flap necrosis and subsequent infections. The prepectoral expander is more susceptible to compression of the skin flap blood circulation, resulting in necrosis and infection because it lacks the protection of the pectoralis major muscle.^45–48^ Many prior studies examining optimal antibiotic prophylaxis, including Gahm et al.,^33^ were performed with primarily dual plane or subpectoral placement of expanders; therefore, it may be difficult to compare complication rates with the mostly prepectoral placement of expanders in this study. However, early studies on prepectoral expanders with ADM demonstrated similar complication rates^49^, with infection still as the main cause of reconstruction failure.^45^ The fact that prepectoral expanders ended up being the most common during the course of this study and yet we found infection rates within the noninferiority range for both groups suggests that even more antibiotics beyond 1 week do not provide additional protection, regardless of the plane of placement. A systematic review of complications in prepectoral breast reconstruction demonstrated variable results and overall low quality of evidence.^46^ Therefore, it is timely that our study provides a high level of evidence for complications related to the now more commonly used prepectoral expander. Other variables such as skin-sparing or nipple-sparing techniques, presence or absence of drains, and duration of drain placement can significantly influence SSI risk. The distribution of these variables was similar between the two groups due to the randomized design of the study.

Several studies show almost 31% SSI rates in operations with ADM use,^50,51^ while more recent studies have shown no difference in short term complications (including SSI) with ADM use in TE-BR.^47^ Our subgroup analysis with and without ADM use did not show higher SSI rates in the ADM group, though the *p*-values were not significant. Additionally, this RCT did not demonstrate a difference in complications related to the type of skin prep, irrigation, or drain dressing, suggesting that these surgical care factors do not play a significant role in postoperative infections.

There are limitations to our study. Achieving the proposed sample size was restricted by the pace of patient accrual. While beyond the scope of this paper, these issues were magnified as the study extended across the entire COVID-19 period. The pandemic exerted a large impact on sites’ ability to initiate their trials and to assess and enroll patients. Furthermore, accrual efforts were diminished by the increased popularity of the direct-to-permanent-implant technique, an exclusion criterion for this RCT. Despite these difficulties, this effort represents one of the largest multi-institutional RCTs ever reported in plastic surgery literature.

## Conclusions

In a multi-institutional RCT, expander-based breast reconstruction patients with either a single preoperative antibiotic dose or with an additional 1 week of antibiotics prophylaxis have comparable SSI rates, hospital readmission, and premature expander removal rates due to SSI. While the findings from this multi-institutional RCT did not demonstrate that a single preoperative dose of antibiotics is not inferior to a 7-day postoperative antibiotic regimen in preventing SSI in immediate TE-BR with statistical significance, there was also no evidence to support the 7-day regimen. Notably, the week-long antibiotic group had later infections, more repeat infections, and the only antibiotic-related complications in the study. Given these findings, our study supports limiting the use of prophylactic antibiotics to match CDC guidelines. Our study also continues to demonstrate the importance of the timing of follow-up of these patients beyond 30 days post operation, which is typically the limit of large surgical databases. This Level I study adds to the growing body of research examining the optimal antibiotic prophylaxis after expander-based breast reconstruction.

## Electronic supplementary material

Below is the link to the electronic supplementary material.

Supplementary file1 (PDF 175 KB)

Supplementary file2 (PPTX 45 KB)

Supplementary file3 (DOCX 26 KB)

Supplementary file4 (DOCX 25 KB)

Supplementary file5 (DOCX 17 KB)

Supplementary file6 (DOCX 15 KB)

Supplementary file7 (DOCX 15 KB)

Supplementary file8 (DOCX 14 KB)

Supplementary file9 (DOCX 16 KB)
