# Supplementary material for: Antibiotics and Surgical Site Infection in Expander-Based Breast Reconstruction Trial (ASSERT)
Source: Ann Surg Oncol. 2025 Oct 14;33(4):3033–44. doi: 10.1245/s10434-025-18472-6 (PMC12982282; doi:10.1245/s10434-025-18472-6)
Supplement: Supplementary file 1 — Supplementary file1 (PDF 175 KB) [file 10434_2025_18472_MOESM1_ESM.pdf]

# A Multi-institutional Randomized Controlled Trial to Determine the Optimal Antibiotic Prophylaxis for Tissue Expander-based Breast Reconstruction


**CO-PRINCIPAL INVESTIGATOR:** **Christine Rohde, MD, MPH**
Columbia University
Phone: 212-342-3707
Email: chr2111@cumc.columbia.edu

**CO-PRINCIPAL INVESTIGATOR:** **Brian Gastman, MD**
Cleveland Clinic, Ohio
Phone: 216-444-2501
Email: gastmab@ccf.org

**COORDINATING CENTER:** **The Plastic Surgery Foundation**
444 East Algonquin Road
Arlington Heights, IL 60005
Phone: 847-228-3358
Email: skaur@plasticsurgery.org



**FUNDING AGENCY:** **The Plastic Surgery Foundation**

**TABLE OF CONTENTS**

**1.0 Introduction** ..... 3
1.1 Background ..... 3
1.2 Rationale ..... 4

**2.0 Study Objectives** ..... 5

**3.0 Selection of Subjects** ..... 6
3.1 Inclusion Criteria ..... 6
3.2 Exclusion Criteria ..... 6

**4.0 Subject Screening and Enrollment** ..... 6

**5.0 Study Design & Methods** ..... 6
5.1 Study Design ..... 7
5.2 Study Procedures ..... 7
5.3 Subject Withdrawal ..... 9
5.4 Compensation ..... 10
5.5 Future Directions ..... 10

**6.0 Statistical Analysis** ..... 10

**7.0 Data Collection, Record Keeping & Privacy Protection** ..... 13

**8.0 Risks and Benefits** ..... 14

**9.0 References** ..... 14

## 1.0 INTRODUCTION

## 1.1 BACKGROUND

Among plastic surgeons, the clinical practice of prescribing postoperative prophylactic antibiotics following postmastectomy breast reconstruction with tissue expanders ranges widely from no postoperative antibiotics, to 5-7 days post-op, to antibiotics until the drains are removed.[1] The Centers for Disease Control and Prevention (CDC) recommends a single preoperative antibiotic dose for clean and clean-contaminated procedures, even in the presence of a drain.[2] In their clinical practice guideline for antimicrobial prophylaxis, the American Society of Health-System Pharmacists (ASHP), the Infectious Diseases Society of America (IDSA), the Surgical Infection Society (SIS), and the Society for Healthcare Epidemiology of America (SHEA) recommended that "antimicrobial prophylaxis should be limited to less than 24 hours, regardless of the presence of indwelling catheters or drains".[3]

Unnecessary administration of antibiotics leads to the development of bacterial resistance and adverse events, such as nausea/vomiting, rash, *Clostridium difficile* infections, acute kidney injury, and others.[4] Consequently, there is an emphasis on using antibiotics for prophylactic indications only when supported by evidence-based medicine. A recent retrospective study found that the implementation of SCIP protocols and the resulting 24-hour limited post-op antibiotic course was associated with a significant increase in tissue expander reconstruction infections, compared with historical controls of giving antibiotics until the drains were removed.[5] A systematic review of 13 studies suggested that prolonged antibiotic use after breast reconstruction surgery was associated with a decrease in SSI risk (pooled RR = 0.508).[6] In contrast, a retrospective market database study of over 7000 patients found no difference in surgical site infections or prosthesis explantation between patients who took post-operative antibiotics and those who did not[7] Additionally, a noninferiority randomized control trial in 112 patients undergoing tissue expander breast reconstruction with acellular dermal matrix (ADM) found no significant difference between a 24-hour or prolonged course of antibiotics.[8] This study used a clinically wide noninferiority margin of 12%+/- 10%, implying that an infection rate that differed from 2% to 22% between the two groups would still be considered statistically noninferior. This wide margin enabled a smaller sample size but is arguably too wide for clinical significance. None of these studies have provided sufficient or conclusive evidence to affect antibiotic practice patterns among plastic surgeons on a large-scale. Surgical site infection (SSI) is likely the most common complication in all of surgery, with major consequences to patients' mental and physical outcomes, as well as contributing to rising healthcare costs. Plastic surgery, in particular, with its frequent use of implanted alloplastic materials, has a higher risk of SSI compared with non- implant-based surgeries. In tissue expander breast reconstruction, the reduced vascularity of the soft tissue envelopes that plastic surgeons place implants within versus other specialties (e.g. orthopedic surgery) places these implants at even higher risk of infection and exposure. Infection and loss of an implant often necessitate the use of secondary surgical options, delay definitive reconstruction, and possibly interfere with timing of adjuvant

cancer therapies. Preventing SSI can reduce the need for subsequent antibiotics, hospitalizations, and secondary surgeries that lead to poorer patient outcomes and higher cost of care. Because of these concerns, most plastic surgeons use a course of post-operative prophylactic antibiotics after tissue expander breast reconstruction.

## 1.2 RATIONALE

With the CDC creating a national action plan to reduce unnecessary prophylactic antibiotics for clean non-contaminated cases by advocating use of only a single pre-operative dose of antibiotics (see: https://www.cdc.gov/drugresistance/pdf/national_action_plan_for_combating_antibotic-resistant_bacteria.pdf ) there is mounting pressure on the plastic surgery community to adopt this regimen of SSI prophylaxis. Because there is no definitive study addressing this issue, plastic surgeons use a wide variety of protocols for their SSI prophylaxis, especially in implant based breast reconstruction.[1,9] Most published prospective studies have occurred in one or two institutions and/or had relatively small sample sizes.[8,10,11] Only a few studies exist that attempt to determine the optimal prophylactic antibiotic regimen for tissue expander breast reconstruction (TE-BR).[5-8,13-16] Randomized, noninferiority trials comparing antibiotic regimens for caesarean sections and cholecystectomy operations are being performed, but neither of these operations include foreign bodies or devitalized soft tissue.[17,18] Plastic surgeons have generally not adopted CDC guidelines because these recommendations were not based on studies in plastic surgery patients, and the use of foreign body implants underneath devitalized soft tissue can be associated with higher infection risks. Based on the literature and from self-reporting from ASPS membership, there is a wide range of prophylactic antibiotic use to prevent SSI from as little as one preoperative dose to many weeks of therapy; one week of post-operative antibiotics is the most common form. However, the Centers for Disease Control (CDC) recommends a single preoperative dose of antibiotics for clean cases, even in the presence of a drain. Prolonged antibiotic courses can lead to antibiotic complications and development of resistance.

Definitive studies to determine an optimal therapeutic strategy to prevent SSI in implant-based plastic surgical procedures are lacking. This study will address a dilemma in common plastic surgical operation: the use of post-operative antibiotics in prosthesis-based breast reconstruction. The goal of this Phase III multi- institutional prospective randomized control trial is to determine whether the CDC recommended single pre- operative dose of antibiotics is not inferior to one-week of postoperative prophylactic antibiotics in preventing surgical site infection (SSI) following postmastectomy breast reconstruction with tissue expanders. The proposed multi-institutional randomized controlled trial would provide the strongest level of evidence and allow ASPS to make the strongest recommendation regarding peri-operative antibiotic regimens. Randomization minimizes bias and increases reliability of the results.

## 2.0 STUDY OBJECTIVES

A randomized controlled trial comparing prophylactic antibiotic regimens in tissue expander breast reconstruction would provide direct evidence for the appropriate duration of antibiotics in these situations. **Our central hypothesis is that a single pre-operative dose of intravenous antibiotics with intraoperative redosing (SPD) is no worse (noninferiority design) at preventing SSI in tissue expander-based breast reconstruction (TE-BR) than an additional week of post-operative antibiotic prophylaxis (WPO).** To test this hypothesis, we will perform this study with following aims:

**Aim 1. To evaluate the efficacy of single preoperative dose versus one-week antibiotic regimens in preventing surgical site infection after tissue expander breast reconstruction, in a noninferiority randomized controlled trial.** *We hypothesize that the SSI rate will not differ by more than 6%, suggesting that additional antibiotic prophylaxis is unnecessary for TE-BR.* The plastic surgery literature is based mainly on retrospective analyses and small trials, with some studies showing that SPD is as good as additional post- operative antibiotics to prevent SSI, and others showing that it is inadequate. In this study, we will first assess, from a multi-institutional RCT, the rates of SSI from the SPD vs. the WPO groups. We will then assess the type, duration and method (oral vs. intravenous) of subsequent antibiotic use for patients who develop SSIs in each group.

**Aim 2. To determine sequela of SSI in the SPD and WPO groups in a RCT alloplastic breast reconstruction study.** *We hypothesize that the two groups will have comparable readmission and premature expander removal rates due to SSI. We hypothesize that the WPO group will have more instances of adverse antibiotic side effects compared with the SPD group.* We will first compare the ER visit and 30- and 90-day hospitalization rates between these two groups. We will assess length of hospitalization, need for surgery, extent of surgery (e.g. wound revision, loss of implant), and speciation of any cultures from the wound or tissues. We will also look for antibiotic side effects in each group and assess if any secondary infections from the two groups differed, including resistant bacterial strains and yeast infections.

This RCT has the potential to change surgical practice to optimize the best prophylactic measures to prevent SSI in a commonly performed operation in plastic surgery. It would also establish necessary infrastructure for conducting RCTs, to bring the best level of evidence-based medicine to plastic surgery.

## 3.0 SELECTION OF SUBJECTS

### 3.1 INCLUSION CRITERIA

Women 18 years or older, undergoing unilateral or bilateral mastectomy (including breast cancer, any stage, or prophylactic) and immediate tissue expander reconstruction (including submuscular, submuscular and ADM, or pre-pectoral placement).

The clinical care team for each patient will determine whether she is a candidate for mastectomy and tissue expander breast reconstruction, and therefore eligible for this trial. Institutional study teams will then go over inclusion/exclusion criteria prior to enrollment.

### 3.2 EXCLUSION CRITERIA

- Breast cancer patients not undergoing mastectomy
- Patients undergoing direct-to-implant reconstruction
- Patients undergoing delayed reconstruction
- Patients having autologous reconstruction
- History of radiation to the breast or chest
- History of previous breast reconstruction on the side of expander placement
- Patients with serious existing systemic infection, defined as 2 or more of the following: Peripheral body temperature >38 degrees Celsius CRP >5g/L Leukocytes > 12,000/microliter at the time of enrollment.

## 4.0 SUBJECT SCREENING AND ENROLLMENT

Patients who are about to undergo mastectomy and tissue expander-based reconstruction will be screened for eligibility by participating surgeons and study personnel based on inclusion/exclusion criteria. Upon passing these criteria and based on patient agreement to participate, patients will sign the study informed consent form with the research staff. All documents related to eligibility will be properly stored via the appropriate data safety manager at each site.

## 5.0 STUDY DESIGN & METHODS

### 5.1 STUDY DESIGN

This study is a Phase III prospective, multi-institutional, noninferiority, randomized control trial comparing two prophylactic antibiotic dose regimens in patients receiving immediate breast reconstruction with tissue expanders. Our central hypothesis is that a single pre-operative dose of intravenous antibiotics with intraoperative redosing (SPD) is no worse (noninferiority design) at preventing SSI in tissue expander-based breast reconstruction (TE-BR) than an additional week of post-operative antibiotic prophylaxis (WPO).

## 5.2 STUDY PROCEDURES

After enrolling a patient in study, the site's study coordinator will be able to randomize the patient to either the SPD or WPO group using the randomization module that will be set up in the REDCap database. Based on the design of this study, neither the surgeon nor patient will be blinded to allocation. The site will proceed with surgery, and data points in the protocol and as outlined in the case report form (CRF), will be documented.

For eligible patients, the following patient characteristics and operative data will be recorded:

- Age
- Height
- Weight
- Bra cup size
- Smoking Status
- Co-morbidities
- Operating Center and facility type
- Date of operation
- Duration of surgery
- Number of intraoperative antibiotic doses
- Type of skin prep
- Type of pocket irrigation
- Chemotherapy (type, timing e.g. neoadjuvant or adjuvant)
- Mastectomy type: Nipple or Skin sparing Mastectomy weight
- Post-operative radiation
- Length of skin flaps from their base
- Expander type
- Position of Expander (Pre-pectoral, Subpectoral)
- Intraoperative expansion volume
- Number of expansions
- Type of drain dressing
- Number of drains
- Implant type
- Use and type of Acellular Dermal Matrices (ADM)
- Laterality of mastectomy/ expander/implant/ ADM
- Axillary lymphadenectomy
- Vascular imaging status (e.g. fluorescence indocyanine green imaging)
- Date of drain removal
- Date of expander exchange

- Date of infection onset
- Antibiotic type used for SSI treatment
- Date of TE removal for infection

**Surgical standardization:**
Intravenous cefazolin, a cephalosporin antibiotic, will be prescribed to all patients before surgery. In case of allergy to cefazolin, intravenous clindamycin will be prescribed. For allergy to both antibiotics, an appropriate antibiotic should be chosen by the surgeon and documented. All patients will receive one dose of antibiotic within 60 minutes prior to incision, and any intraoperative doses of antibiotics according to current recommendations based on the antibiotic's dosing and on operative time. Skin preparation will be performed using chlorhexidine-based sterilization[19] or according to surgeon's standard practice and documented. The other operative details should proceed according to the surgeon's standard procedure. Post-operative checks and follow-up will also proceed according to surgeon's standard practice, but all patients will have their first post-operative visit by two weeks post-op at the latest, and a second visit within 30 days post-op (+/- 7 days).

The patients assigned to receive one week of post-operative antibiotics will be prescribed first generation Cephalosporin of surgeon's choice. Outcomes as outlined below will be recorded on the CRF and reported to the central study database. At the first post-operative visit, patients assigned to receive one week of post-operative antibiotics will be asked to report which doses they took and to return the bottle and any unused pills when completed. Study personnel will document if, when and why patients stopped taking antibiotics prior to completion of the course. As discussed in the statistical plan, analysis will be by both intent-to-treat and per-protocol.

**Primary Endpoint:** Surgical site infection as defined by CDC criteria within 30 days after the index procedure

The Surgical Site Infection (SSI)/ Superficial incisional SSI must meet the following criteria:

Date of event occurs within 30 days after any National Healthcare Safety Network (NHSN) operative procedure (where day 1 = the procedure date) AND involves only skin and subcutaneous tissue of the incision AND patient has at least one of the following:

- purulent drainage from the superficial incision.
- organism(s) identified from an aseptically-obtained specimen from the superficial incision or subcutaneous tissue by a culture or non-culture based microbiologic testing method which is performed for purposes of clinical diagnosis or treatment (for example, not Active Surveillance Culture/Testing (ASC/AST)).
- superficial incision that is deliberately opened by a surgeon, physician* or physician

designee and culture or non-culture based testing of the superficial incision or subcutaneous tissue is not performed AND patient has at least one of the following signs or symptoms: localized pain or tenderness; localized swelling; erythema; or heat.

- diagnosis of a superficial incisional SSI by a physician* or physician designee.

* The term physician for the purpose of application of the NHSN SSI criteria may be interpreted to mean a surgeon, infectious disease physician, emergency physician, other physician on the case, or physician's designee (nurse practitioner or physician's assistant).

For the primary endpoint, a unilateral or bilateral infection will be considered a single per-patient-SSI.

**Secondary Endpoints:**

- Tissue expander or implant removal, per breast versus per patient events,
- Antibiotic and wound culture speciation and
- Antibiotic sensitivities in SSI/deep infections,
- SSI beyond 30 days after index procedure up to time of tissue expander exchange (for an implant or flap),
- Antibiotic-related adverse events, e.g. cholitis, yeast infections, anaphylaxis, etc.

All sites will base an SSI designation on CDC definition and investigator assessment. Despite the CDC guidelines on SSI definition, the clinical diagnosis of SSI occasionally may not be clear-cut. Any SSI determination that is not clear-cut will be reviewed by all investigators to make sure that a majority agree with that diagnosis. Additionally, mastectomy skin flap necrosis can sometimes be the driver for expander removal, rather than an infection. These observations will be accurately recorded in the CRF and will help in determining that an outcome is related to an SSI. Overall, investigator judgment of appropriate clinical care will determine management of post-operative signs and symptoms. Antibiotic side effects will be assessed and documented by the treating team at the first two post-operative visits within 30 days of surgery.

## 5.3 SUBJECT WITHDRAWAL

Patients will be given opportunities to ask questions during enrollment and the study and can decline to participate or drop out of the study at any time. If a participant withdraws, any data acquired prior to study withdrawal may be stored for record keeping purposes.

## 5.4 COMPENSATION

Subjects will not be compensated for participation in this study.

## 5.5 FUTURE DIRECTIONS

We expect to yield critical information about optimization of antibiotic protocols in implant-based breast reconstruction. Regardless of whether or not our hypothesis is proven, these data will be practice changing if they lead to less variability in antibiotic protocols. We will also be able to develop a risk-benefit stratification of the various antibiotic protocols SPD vs. WPO, as a slight increase in infections in one group could be balanced by other secondary negative consequences of antibiotics that could still lead to that approach being clinically superior to the other. Finally, developing a coordinated multicenter RCT under the auspices of the PSF will help develop a precedent for future plastic surgery definitive clinical trials that could be a paradigm shift in the level of evidence required before universal adoption of any practice management algorithm or recommendation.

Because of the design of this multi-institutional RCT, the process and results can facilitate the design of future studies to further optimize antibiotic use in implant-based reconstruction. This study can also help establish the culture and the infrastructure to use the power of randomized controlled trials to bring the highest level of evidence-based medicine to plastic surgery.

## 6.0 STATISTICAL ANALYSIS

This multi-center randomized controlled trial is designed as a noninferiority trial (NI) with a 1:1 allocation ratio between the parallel SPD and WPO groups. The main endpoint of interest is the rate of surgical site infection (SSI) by CDC guidelines within 30 days of surgery; secondary endpoints include the SSI rate before second stage reconstruction, number of hospitalizations, and unplanned expander removal prior to exchange surgery. From recent studies, the infection rate in immediate breast reconstruction with and without acellular dermal matrix has a wide range, with most between 5%-19%.[21-23] Based on this evidence, the point estimate for post-operative rate of SSIs is set at 12%.

### Noninferiority Design

A noninferiority design randomized clinical trial is appropriate for this study that compares a medication regimen with an active comparator (in this case, two different regimens of active antibiotic drugs).[24] Non- inferiority clinical trials are preferred when the belief is that the interventions/arms have approximately the same efficacy, but may offer other benefits such as:

better safety profile, lower cost, etc. If we are able to demonstrate noninferiority of a single dose compared with one week of prophylaxis, adverse events related to antibiotic use would argue for adoption of a single dose for antibiotic prophylaxis.

For design of an antibiotic regimen, two arm RCT study with A = single dose and B = single dose plus seven days of postoperative doses, there are 3 potential outcomes:

1) (Superiority) A superior to B in preventing surgical site infections
   With the exception of adverse events, it is not clinically intuitive to assume that fewer antibiotics will yield fewer infections.
2) (Superiority) B superior to A
   This outcome is clinically intuitive and the reason why many surgeons favor this regimen without evidence of superiority. However, it is neither a compelling clinical question to study what already general practice is, nor is it in line with CDC guidelines for other surgeries, which promote the single dose regimen.
3) (Noninferiority) A is not worse than B
   This is a compelling clinical question and if true, will be both practice management changing and would allow for plastic surgery implant-based surgery to align with CDC guidelines in general.

Additionally, if noninferiority cannot be established (null hypothesis cannot be rejected) at the conclusion of the trial, the trial is designed and powered to formally conduct a superiority test.[25] Therefore, under both scenarios, we will be able to make a clinically and statistically significant conclusion at the completion of the study.

Randomization: patients will be assigned to one of the two arms using varying block size randomization within each site to ensure balance allocation.
Blinding: not applicable
Accrual: We expect to accrue all patients within one and a half years across five study sites, with an additional 6 months for unexpected delays.

**Power/sample size calculations** are based on estimates from previous studies and considered 12% vs. 18% SSI rate for the post-operative antibiotic (reference group) and CDC recommendations (under the null hypothesis of inferiority), respectively. We plan a noninferiority design with a clinically meaningful noninferiority margin of $\delta$=6% between the group proportions. Estimates were computed using one-sided Z test (un-pooled), with significance level targeted at 0.025. To conclude noninferiority of the CDC recommended single dose, compared with the post-operative antibiotic course, at 80% power, a total of 922 patients would be needed (461 per arm). Accounting for dropouts, we would plan to recruit 500 patients per group (1000 total). This sample size will also allow us to assess for superiority, if noninferiority cannot be established at the conclusion of the study. We will also compute the

95% confidence interval (CI) for the difference in proportions between the two arms. Noninferiority will be established if the lower limit of the 95% CI is above –δ (-6%). If the upper bound of the 95% CI is below –δ, then the noninferiority null hypothesis will be rejected, and a superiority test will sequentially be conducted.

**Statistical Analyses:**
Analyses would be by intent-to-treat (ITT) and per protocol (as-treated) (PP) in accordance with Committee on Proprietary Medical Products (CPMP) guidelines for noninferiority trials.[25] Descriptive statistics will be used to summarize continuous (mean ± standard deviation or median –interquartile range) and categorical (frequency, percentages) variables, along with the corresponding 95% confidence intervals (CI). Comparisons between the two groups will be performed using chi-squared/Fisher's Exact tests or t-tests for categorical and continuous variables, respectively. Logistic regression models will be employed to compare the odds of developing any SSI between the two groups (primary analysis). Unadjusted and adjusted analyses (adjusting for patients' characteristics and other observed confounders) will be performed. Since multiple infections are possible for each patient, we will record and model the total number of infections using Poisson regression. Further, if the sample size allows, we plan to conduct subgroup analyses by the type of intervention: unilateral and bilateral mastectomy separately. In order to address inevitable differences/variability in surgeons and interventions, random effects models will be considered in the analysis.

An interim analysis for futility is planned after half the data have been collected in each arm (N=250 out of max sample size of 500 for each arm). Futility will be assessed by computing conditional power, i.e., probability that the final result will be significant, given the data obtained up to the time of the interim look. Given the two SSI rates, $p_1$=12% (reference group) vs. $p_2$=18% (non-inferiority alternative), at 0.025 type I error, we will use Jennison and Turnbull (2000) method to calculate the one-sided conditional power at interim analysis, based on the observed test statistic $z_k$ calculated from the observed data[26]. For example, values of the test statistic $z_k < -2$ would provide high power (over 88%) to indicate that the study is likely to result in useful results. Alternatively, values of $z_k > 0$ would generate low power (~20%), indicating little chance of showing at the end of the trial that the rate of surgical site infection by CDC criteria is non-inferior (futility) to the standard antibiotics regimen. In case of futility, the trial will be stopped after the interim analysis.

The graph below shows the conditional power at interim analysis as a function of various test statistics.

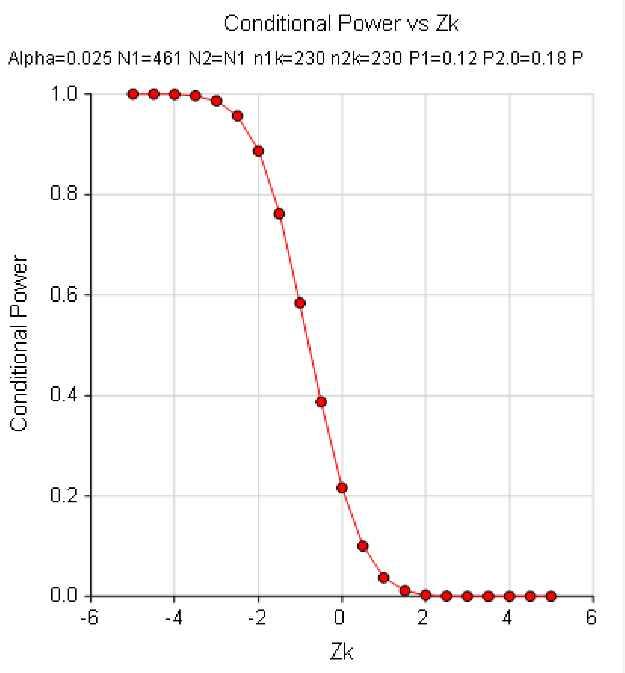


## 7.0 DATA COLLECTION, RECORD KEEPING & PRIVACY PROTECTION

Patient demographic information will be obtained from existing patient records. This data will be collected for research purposes only and not used for any other purposes. Data will be collected prospectively. The risk of breach of confidentiality and loss of privacy will be minimized by using coded data and safely storing all data in password protected devices and locked storage cabinets at the study site. All paper documents will be stored in a locked cabinet. The master file linking the participant's names with the identification number will be kept on a secure server at the study sites and will only be accessible to the site-PI, study coordinator and authorized research personnel.

A central study database will be set up by The Plastic Surgery Foundation, using REDCap. The database will be accessible to the study sites by secure web access. Once the site assigns a patient a study ID number, a research assistant at the site will use this data to populate the central study database. The coded data, devoid of all direct identifiers, except dates, will be sent to The Plastic Surgery Foundation for the development of final study data set and data analysis. Access to the data will only be available to authorized users at each site and The PSF through a secure connection to the server and database. Sites will only have access to their site-specific data, while The PSF will have access to the entire limited dataset.

**Data and Safety Monitoring:** Information regarding patients enrolled in the study will be kept on a password- protected database. Only the investigators of the study will have access to this

information and patient details will be kept under a study ID number, and not under the patient's name. Aggregate data shared between investigators will be coded and de-identified (except dates). This information will be kept on a computer in a locked departmental office at all times.

This study compares two regimens of FDA approved antibiotics being used for their approved purpose and as such, there is no current indication for adverse event monitoring by an independent safety monitoring committee. However, adverse events related to antibiotic use will be recorded by the investigators.

## 8.0 RISKS AND BENEFITS

**Potential Risks and Benefits:** There is no single accepted standard of care for antibiotic prophylaxis in tissue expander breast reconstruction. Since this study compares two regimens of FDA approved antibiotics being used for their approved purpose and all patients will receive the CDC recommended dose of antibiotic prophylaxis before surgery, as well as any recommended doses during surgery, participation in the study does not increase the risk. It is possible that a patient may have a higher or lower risk of surgical site infection, as well as a higher or lower risk of antibiotic side effects depending on the antibiotic regimen. This study seeks to determine if a difference exists, so that future patients can benefit from the optimal duration of antibiotic prophylaxis.

**Protection Against Risk:** All patients will receive the CDC recommended preoperative dose of antibiotic prophylaxis, as well as any recommended intraoperative doses based on operative time. Patients with suspicion for SSI or antibiotic-related adverse event will be treated according to the surgeon's practice and standard of care.

## 9.0 REFERENCES


1. Phillips BT, Wang ED, Mirrer J, et al. Current practice among plastic surgeons of antibiotic prophylaxis and closed-suction drains in breast reconstruction: Experience, evidence, and implications for postoperative care. *Ann Plast Surg*. 2011;66:460–465.
2. Berríos-Torres SI, Umscheid CA, Bratzler DW, et al. Centers for Disease Control and Prevention Guideline for the Prevention of Surgical Site Infection, 2017. *JAMA Surg.* 2017;152(8):784–791.
3. Bratzler DW, Dellinger EP, Olsen KM. et al. Clinical practice guidelines for antimicrobial prophylaxis in surgery. *Am J Health-Sys Pharm*. 2013;70:195-283.
4. Sartelli, M., Duane, T. M., Catena, F., Tessier, J. M., Coccolini, F., Kao, L. S., ... & Mazuski, J. E. Antimicrobial stewardship: a call to action for surgeons. *Surgical infections*, 2016;17(6), 625-631.
5. Clayton JL, Bazakas A, Lee CN, Hultman CS, Halvorson EG. Once is not enough: withholding postoperative prophylactic antibiotics in prosthetic breast reconstruction is associated with an increased risk of infection. *Plast Reconstr Surg*. 2012;130(3):495-

502.
6. Huang N, Liu M, Yu P, Wu J. Antibiotic prophylaxis in prosthesis-based mammoplasty: a systematic review. *Internat J Surg*. 2015;15:31–37.
7. Ranganathan K, Sears ED, Zhong L, Chung TT, Chung KC, Kozlow JH, Momoh AO, Waljee JF. Antibiotic Prophylaxis after Immediate Breast Reconstruction: The Reality of Its Efficacy. *Plast Reconstr Surg*. 2018;141(4):865-877.
8. Phillips, B.T., Fourman, M.S., Bishawi, M. et al. Are prophylactic postoperative antibiotics necessary for immediate breast reconstruction? Results of a prospective randomized clinical trial. *J Am Coll Surg*. 2016; 222: 1116–1124.
9. Ariyan, S., Martin, J., Lal, A., Cheng, D., Borah, G., Chung, K., Conly, J., Havlik, R., Lee, W.P., McGrath, M., Pribaz, J., Young, V.L. Antibiotic Prophylaxis for Preventing Surgical-Site Infection in Plastic Surgery: An Evidence Based Consensus Conference Statement from the American Association of Plastic Surgeons. *Plast Reconstr Surg*. 2015 Jun;135(6):1723-39.
10. McCarthy, C., Lee, C. Halvorson, E., Riedel, E., Pusic, A., Mehrara, B., Disa, J. The Use of Acellular Dermal Matrices in Two-Stage Expander/Implant Reconstruction: A Multicenter, Blinded, Randomized, Controlled Trial. *Plast Reconstr Surg*. 2012 Nov;130(5 Suppl 2):57S-66S.
11. Lee JC, Ishtihar S, Means JJ, Wu J, Rohde CH. In Search of an Ideal Closure Method: A Randomized, Controlled Trial of Octyl-2-Cyanoacrylate and Adhesive Mesh versus Subcuticular Suture in Reduction Mammaplasty. *Plast Reconstr Surg*. 2018 Oct;142(4):850-856.
12. Srinivasa DR, Garvey PB, Qi J, Hamill JB, Kim HM, Pusic AL, Kronowitz SJ, Wilkins EG, Butler CE, Clemens MW. Direct-to-Implant versus Two-Stage Tissue Expander/Implant Reconstruction: 2-Year Risks and Patient-Reported Outcomes from a Prospective, Multicenter Study. *Plast Reconstr Surg*. 2017 Nov;140(5):869-877.
13. McCullough MC, Chu CK, Duggal CS, Losken A, Carlson GW. Antibiotic Prophylaxis and Resistance in Surgical Site Infection After Immediate Tissue Expander Reconstruction of the Breast. *Ann Plast Surg*. 2016 Nov;77(5):501-505.
14. Alderman A, Gutowski K, Ahuja A, Gray D. Postmastectomy Expander Implant Breast Reconstruction Guideline Work Group. ASPS clinical practice guideline summary on breast reconstruction with expanders and implants. *Plast Reconstr Surg*. 2014 Oct;134(4):648e-55e,
15. Weichman KE, Wilson SC, Weinstein AL, Hazen A, Levine JP, Choi M, Karp NS. The use of acellular dermal matrix in immediate two-stage tissue expander breast reconstruction. *Plast Reconstr Surg*. 2012 May;129(5):1049-58.
16. Richardson C, Mattison G, Roring J, et al. The Optimal Duration of Antibiotic Prophylaxis in Plastic Surgery: A Meta-Analysis of 32 Publications. *Health Sci J*. 2016;10:2.
17. Westen EHMN, Kolk PR, Velzen CLV, Unkels R, Mmuni NS, Hamisi AD, et al. Single-dose compared with multiple day antibiotic prophylaxis for cesarean section in low-resource settings, a randomized controlled, noninferiority trial. *Acta Obstet Gynecol Scand*. 2015;94:43–9.
18. Loozen CS, van Santvoort HC, van Geloven AAW, et al. Perioperative antibiotic prophylaxis in the treatment of acute cholecystitis (PEANUTS II trial): study protocol for a randomized controlled trial. *Trials*. 2017;18(1):390.
19. Mimoz O, Lucet JC, Kerforne T, Pascal J, Souweine B, Goudet V et al. Skin antisepsis

with chlorhexidine- alcohol versus povidone iodine-alcohol, with and without skin scrubbing, for prevention of intravascular-catheter-related infection (CLEAN): an open-label, multicenter, randomised, controlled, two-by-two factorial trial. *Lancet*. 2015;386:2069-77.

20. Viola GM, Raad II, Rolston KV. Breast Tissue Expander–Related Infections: Perioperative Antimicrobial Regimens. *Infection Control and Hospital Epidemiology*. 2014;35(1):75–81.
21. Chun YS, Verma K, Rosen H, Lipsitz S, Morris D, Kenney P et al. Implant-based breast reconstruction using acellular dermal matrix and the risk of postoperative complications. *Plast Reconstr Surg*. 2010;125:429-36.
22. Sbitany H, Wang F, Peled AW, Lentz R, Alvarado M, Ewing CA et al. Immediate implant-based breast reconstruction following total skin-sparing mastectomy: defining the risk of preoperative and postoperative radiation therapy for surgical outcomes. *Plast Reconstr Surg*. 2014;134:396-404.
23. Wang, F., Chin, R., Piper, M., Esserman, L., and Sbitany, H. Do prolonged prophylactic antibiotics reduce the incidence of surgical site infections in immediate prosthetic breast reconstruction? *Plast Reconstr Surg*. 2016; 138: 1141–1149
24. Althunian, T. A., de Boer, A., Groenwold, R., & Klungel, O. H. (2017). Defining the noninferiority margin and analysing noninferiority: An overview. *Brit J Clin Pharm*. *83*(8), 1636-1642.
25. Committee for Proprietary Medicinal Products (CPMP) Points to Consider on Switching between Superiority and Noninferiority. *Brit J Clin Pharm*. 2001;52(3):223-228.
26. Jennison, C. and Turnbull, BW. “Group Sequential Methods with Applications to Clinical Trials,” Chapman & Hall/CRC, Boca Raton, 2000.
