# Supplementary material for: Antibiotics and Surgical Site Infection in Expander-Based Breast Reconstruction Trial (ASSERT)
Source: Ann Surg Oncol. 2025 Oct 14;33(4):3033–44. doi: 10.1245/s10434-025-18472-6 (PMC12982282; doi:10.1245/s10434-025-18472-6)
Supplement: Supplementary file 2 — Supplementary file2 (PPTX 45 KB) [file 10434_2025_18472_MOESM2_ESM.pptx]

## Slide 1
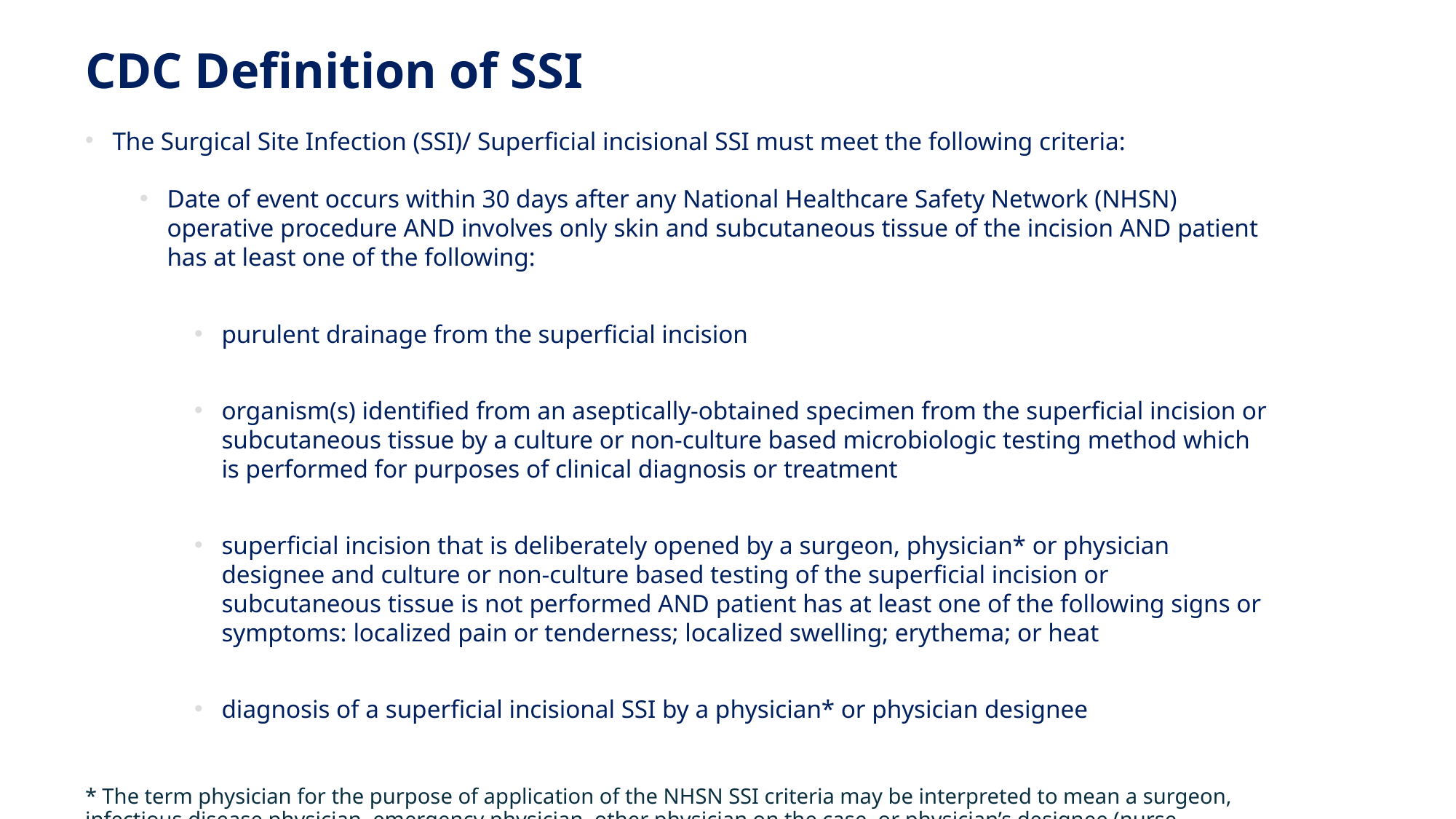

# CDC Definition of SSI
The Surgical Site Infection (SSI)/ Superficial incisional SSI must meet the following criteria:
Date of event occurs within 30 days after any National Healthcare Safety Network (NHSN) operative procedure AND involves only skin and subcutaneous tissue of the incision AND patient has at least one of the following:
purulent drainage from the superficial incision
organism(s) identified from an aseptically-obtained specimen from the superficial incision or subcutaneous tissue by a culture or non-culture based microbiologic testing method which is performed for purposes of clinical diagnosis or treatment
superficial incision that is deliberately opened by a surgeon, physician* or physician designee and culture or non-culture based testing of the superficial incision or subcutaneous tissue is not performed AND patient has at least one of the following signs or symptoms: localized pain or tenderness; localized swelling; erythema; or heat
diagnosis of a superficial incisional SSI by a physician* or physician designee
* The term physician for the purpose of application of the NHSN SSI criteria may be interpreted to mean a surgeon, infectious disease physician, emergency physician, other physician on the case, or physician’s designee (nurse practitioner or physician’s assistant).
