# Supplementary material for: Antibiotics and Surgical Site Infection in Expander-Based Breast Reconstruction Trial (ASSERT)
Source: Ann Surg Oncol. 2025 Oct 14;33(4):3033–44. doi: 10.1245/s10434-025-18472-6 (PMC12982282; doi:10.1245/s10434-025-18472-6)
Supplement: Supplementary file 3 — Supplementary file3 (DOCX 26 KB) [file 10434_2025_18472_MOESM3_ESM.docx]

**Tables A-C, Supplementary Digital Content 3: Operative and Treatment Details**

**A: Operative and Treatment Details**

| **Characteristic** | **SPD n=102** | **WPO n=112** | **p Value** |
| --- | --- | --- | --- |
| **Surgical Facility type** |  |  | 0.207 |
| Main Hospital/ Operating Room, n (%) | 78 (76.5) | 77 (68.8) |  |
| Ambulatory Surgery Center, n (%) | 24 (23.5) | 35 (31.3) |  |
| **Breast Cancer Status** |  |  | 0.144 |
| Invasive, n (%) | 70 (68.6) | 60 (53.6) |  |
| DCIS, n (%) | 18 (17.6) | 29 (25.9) |  |
| Other, n (%) | 3 (2.9) | 7 (6.3) |  |
| None, n (%) (including high risk/ genetic susceptibility) | 11 (10.8) | 16 (14.3) |  |
| **Laterality** |  |  | 0.854 |
| Bilateral Mastectomy, n (%) | 72 (70.6) | 80 (71.4) |  |
| Unilateral Mastectomy (left+right), n (%) | 30 (29.4) | 32 (28.6) |  |
| **Mastectomy Type** |  |  | 0.994 |
| Skin Sparing, n (%) | 48 (47.1) | 53 (47.3) |  |
| Nipple Sparing, n (%) | 38 (37.3) | 41 (36.6) |  |
| Other, n (%) | 16 (15.7) | 18 (16.1) |  |
| **Prophylactic Mastectomy** | 53 (52.0) | 55 (49.1) | 0.677 |
| Unilateral Prophylactic Mastectomy (left+right), n (%) | 44 (43.1) | 37 (33.1) |  |
| Bilateral Prophylactic Mastectomy, n (%) | 9 (8.8) | 18 (16.1) |  |
| **Intraoperative Antibiotic Used** |  |  | 0.895 |
| cefazolin, n (%) | 89 (87.3) | 100 (89.3) |  |
| clindamycin, n (%) | 11 (10.8) | 10 (8.9) |  |
| Other, n (%) | 2 (2.0) | 2 (1.8) |  |
| **ADM Use** |  |  |  |
| ADM used, n (%) Yes | 77 (75.5) | 84 (75.0) | 0.934 |
| **Laterality of ADM use** |  |  | 0.770 |
| ADM used Bilateral, n (%) | 50 (49) | 58 (51.8) |  |
| ADM used Unilateral, n (%) | 27 (26.5) | 23 (20.5) |  |
| **Type of ADM** |  |  | 0.881 |
| Perforated ADM, n (%) | 54 (54) | 60 (53.6) |  |
| Non-perforated ADM, n (%) | 16 (15.7) | 21 (18.8) |  |
| **Position of Expander** |  |  | 0.973 |
| Pre-pectoral with ADM, n (%) | 73 (71.6) | 82 (73.2) |  |
| Pre-pectoral without ADM, n (%) | 19 (18.6) | 20 (17.9) |  |
| Complete submuscular without ADM, n (%) | 3 (2.9) | 3 (2.7) |  |
| Submuscular with ADM, n (%) | 6 (5.9) | 5 (4.5) |  |
| **Intraoperative TE fill** |  |  | 0.623 |
| Air, n(%) | 61 (59.8) | 62 (55.4) |  |
| Saline, n (%) | 14 (13.7) | 20 (17.9) |  |
| Both, n(%) | 1 (1.0) | 1 (0.9) |  |
| None, n (%) | 26 (25.5) | 27 (24.1) |  |
| **Imaging** |  |  | 0.291 |
| Vascular Imaging Done, n (%) yes | 27 (26.5) | 38 (33.9) |  |
| Vascular Imaging Done, n (%) no | 72 (70.6) | 72 (64.3) |  |
| **For WPO Arm, Antibiotic prescribed** |  |  | n/a |
| cephalexin, n (%) | 0 (0) | 48 (42.9) |  |
| cefadroxil, n (%) | 0 (0) | 32 (28.6) |  |
| other, n (%) | 0 (0) | 31 (27.7) |  |
| **Antibiotic course completed for WPO arm** |  |  | n/a |
| Antibiotic course completed for WPO arm, n (%) yes | 0 (0) | 104 (92.9) |  |
| Antibiotic course completed for WPO arm, n (%) no | 0 (0) | 8 (7.1) |  |
| **Post operative Chemotherapy** |  |  | 0.867 |
| Post operative Chemotherapy, n (%) yes | 13 (12.7) | 12 (10.7) |  |
| Post operative Chemotherapy, n (%) no | 82 (80.4) | 94 (83.9) |  |
| Post operative Chemotherapy, n (%) unknown | 5 (4.9) | 5 (4.5) |  |
| **Post operative Radiation** |  |  |  |
| Post operative Radiation, n (%) yes | 14 (13.7) | 22 (19.6) | 0.499 |
| Post operative Radiation, n (%) no | 81 (79.4) | 84 (75.0) |  |
| Post operative Radiation, n (%) unknown | 6 (5.9) | 6 (5.4) |  |

**B. Operative Details**

| **Characteristic** | **SPD n=102** | **WPO n=112** | **P Value** |
| --- | --- | --- | --- |
| **Other Procedures during BR procedure** | 5 (4.9) | 7 (6.3) | 0.520 |
| Sentinel lymph node biopsy only, n (%) | 72 (70.6) | 69 (61.6) |  |
| Axillary Dissection, n (%) | 15 (14.7) | 19 (17) |  |
| None, n (%) | 14 (13.7) | 23 (20.5) |  |
| **Type of Skin Prep** |  |  |  |
| Chloraprep, n (%) | 73 (71.6) | 81 (72.3) | 0.903 |
| Betadine, n (%) | 21 (20.6) | 19 (17.0) | 0.497 |
| Chlorhexidine, n (%) | 12 (11.8) | 12 (10.7) | 0.808 |
| Other, n (%) | 1 (1.0) | 0 (0.0) | 0.294 |
| **Type of Pocket Irrigation** |  |  |  |
| Triple Antibiotics, n (%) | 46 (45.1) | 45 (40.2) | 0.467 |
| Saline, n (%) | 35 (34.3) | 24 (21.4) | 0.035 |
| Betadine, n (%) | 18 (17.6) | 18 (16.1) | 0.758 |
| Bacitracin, n (%) | 1 (1.0) | 0 (0.0) | 0.294 |
| Other, n (%) | 31 (30.4) | 40 (35.7) | 0.409 |
| **Type of Drain Dressing** |  |  |  |
| Biopatch, n (%) | 26 (25.5) | 24 (21.4) | 0.483 |
| Drain dressing antibiotic ointment, n (%) | 8 (7.8) | 12 (10.7) | 0.471 |
| Dry gauze, n (%) | 5 (4.9) | 7 (6.3) | 0.669 |
| CHG dressing, n (%) | 6 (5.9) | 4 (3.6) | 0.424 |
| Xeroform, n (%) | 4 (3.9) | 6 (5.4) | 0.619 |
| Other, n (%) | 35 (34.3) | 34 (30.4) | 0.536 |
| None, n (%) | 27 (26.5) | 34 (30.4) | 0.529 |

**C. Operative and Treatment Details**

| **Operation Details** | **SPD** | **WPO** | **p Value** |
| --- | --- | --- | --- |
| Number of Intraoperative Antibiotic doses Range, mean (SD), n | 1-3, 1.465 (.557), n=101 | 0.5-3, 1.527 (.625), n=112 | 0.452 |
| Time added to surgery for ancillary procedures in hours, Range, mean (SD), n | 0.25-4, 2.54 (1.8), n=4 | 0.97-8, 4.99 (3.6), n=3 | 0.285 |
| Duration of Surgery in hours Range, mean (SD), n | 1.22-10, 4.13 (1.47), n=102 | 1.32-9, 4.2 (1.56), n=112 | 0.701 |
| **Drains** |  |  |  |
| Number of drains, Range, mean (SD), n | 1-5, 2.46 (1.12), n=102 | 1-4, 2.54 (1.08), n=112 | 0.714 |
| Time to drain removal days, Range, mean (SD), n | 5-41, 13.35 5.745), n=94 | 4-78, 15.31(10), n=104 | 0.301 |
| **TE Size** |  |  |  |
| Left TE Size mL, Range, mean (SD), n | 250-800, 490 (129), n=90 | 300-900, 510 (147), n=96 | 0.323 |
| Right TE Size mL, Range, mean (SD), n | 250-800, 495 (125), n=83 | 250-900, 513 (152), n=94 | 0.396 |
| Left Initial Fill Volume mL, Range, mean (SD), n | 20-400, 187 (116), n=68 | 10-650, 194 (138), n=68 | 0.753 |
| Right Initial Fill Volume mL, Range, mean (SD), n | 10-400, 175 (122), n=61 | 10-650, 183 (137), n=68 | 0.709 |
| Average number of expansions, Range, mean (SD), n | 0-11, 2.9 (1.9), n=91 | 0-10, 2.7 (1.99), n=97 | 0.468 |
| Left Mastectomy Weight g, Range, mean (SD), n | 117-1500, 586 (323), n=69 | 100-1795, 561 (331), n=73 | 0.648 |
| Right Mastectomy Weight g, Range mean (SD), n | 107-1400, 593 (308), n=65 | 77-1749, 536 (331), n=73 | 0.297 |
| **Chemotherapy** |  |  |  |
| Post operative Chemotherapy duration in days Range, mean (SD), n | 63-147, 107 (36.2), n=4 | 65-268, 132.25 (92), n=4 | 0.628 |
| Post operative Radiation therapy duration in days Range, mean (SD), n | 32-69, 42.1 (13.8), n=7 | 31-70, 40.5 (11.1), n=14 | 0.771 |
