# Supplementary material for: Antibiotics and Surgical Site Infection in Expander-Based Breast Reconstruction Trial (ASSERT)
Source: Ann Surg Oncol. 2025 Oct 14;33(4):3033–44. doi: 10.1245/s10434-025-18472-6 (PMC12982282; doi:10.1245/s10434-025-18472-6)
Supplement: Supplementary file 4 — Supplementary file4 (DOCX 25 KB) [file 10434_2025_18472_MOESM4_ESM.docx]

**Table, Supplementary Digital Content 4: Endpoint Analysis by ADM Use**

| **Endpoints** | **Non-ADM Users** | | | **ADM Users** | | |
| --- | --- | --- | --- | --- | --- | --- |
|  | **SPD**  **(N=25)** | **WPO**  **(N=28)** | **P-Value** | **SPD**  **(N=77)** | **WPO**  **(N=84)** | **P-Value** |
| SSI: Infection within 30 days(n%) | 3 (12%) | 5 (18%) | .114 | 14 (18%) | 7 (8%) | .766 |
| Infection within 90 days (n%) | 5 (20%) | 6 (21%) | .253 | 18 (23%) | 12 (14%) | .693 |
| Infection within 180 days (n%) | 6 (24%) | 8 (29%) | .192 | 18 (23%) | 12 (14%) | .693 |
|  |  |  |  |  |  |  |
| TE removal for infection within 30 days | 1 (4%) | 0 (0%) |  | 5 (7%) | 2 (2%) |  |
| Any TE Removal for infection | 6 (24%) | 5 (18%) |  | 12 (16%) | 8 (10%) |  |
|  |  |  |  |  |  |  |
| **Additional endpoints** |  |  |  |  |  |  |
| Return to OR within 30 days, n(%) | 4 (16%) | 3 (11%) |  | 7 (9%) | 8 (10%) |  |
| Hospitalization within 30 days, n(%) | 1 (4%) | 4 (14%) |  | 8 (10%) | 3 (4%) |  |
| Hospitalization between 31-90 days, n(%) | 3 (12%) | 4 (14%) |  | 3 (4%) | 4 (5%) |  |
|  |  |  |  |  |  |  |
| **If yes to Infection (N=44)** | **SPD**  **(N=6)** | **WPO**  **(N=8)** |  | **SPD**  **(N=18)** | **WPO**  **(N=12)** |  |
| Intravenous Antibiotic, n(%) | 5 (83%) | 5 (63%) |  | 8 (44%) | 4 (33%) |  |
| Oral Antibiotic, n(%) | 4 (67%) | 7 (88%) |  | 14 (78%) | 8 (67%) |  |
| Surgical Intervention, n(%) | 6 (100%) | 6 (75%) |  | 10 (56%) | 8 (67%) |  |
| Close Observation only, n(%) | 0 (0%) | 0 (0%) |  | 0 (0%) | 0 (0%) |  |

^*^P-value was corresponding to test the null hypothesis that P_WPO_ - P_SPD_  ≤ -0.06 for the infection related endpoints

where P_WPO_ and P_SPD_ are the true probability of infection for WPO and SPD group respectively. P-values for the remaining

analyses were not provided as the margin of inferiority was not pre-specified.
