# Supplementary material for: Antibiotics and Surgical Site Infection in Expander-Based Breast Reconstruction Trial (ASSERT)
Source: Ann Surg Oncol. 2025 Oct 14;33(4):3033–44. doi: 10.1245/s10434-025-18472-6 (PMC12982282; doi:10.1245/s10434-025-18472-6)
Supplement: Supplementary file 5 — Supplementary file5 (DOCX 17 KB) [file 10434_2025_18472_MOESM5_ESM.docx]

**Table, Supplementary Digital Content 5: Endpoint Analysis by Position of Expander (Pre-pectoral versus Subpectoral)**

| **Endpoints** | **Submuscular** | | | **Pre-Pectoral** | | |
| --- | --- | --- | --- | --- | --- | --- |
|  | **SPD**  **(N=9)** | **WPO**  **(N=7)** | **P-Value** | **SPD**  **(N=92)** | **WPO**  **(N=103)** | **P-Value** |
| SSI: Infection within 30 days(n%) | 3 (33%) | 0 (0%) | .918 | 14 (15%) | 12 (12%) | .309 |
| Infection within 90 days (n%) | 3 (33%) | 0 (0%) | .918 | 20 (22%) | 18 (18%) | .380 |
| Infection within 180 days (n%) | 3 (33%) | 0 (0%) | .918 | 21 (23%) | 20 (19%) | .329 |
|  |  |  |  |  |  |  |
| TE removal for infection within 30 days | 1 (11%) | 0 (0%) |  | 5 (5%) | 2 (2%) |  |
| Any TE Removal for infection | 2 (22%) | 0 (0%) |  | 16 (17%) | 13 (13%) |  |
|  |  |  |  |  |  |  |
| **Additional endpoints** |  |  |  |  |  |  |
| Return to OR within 30 days, n(%) | 1 (11%) | 0 (0%) |  | 10 (11%) | 10 (10%) |  |
| Hospitalization within 30 days, n(%) | 2 (22%) | 0 (0%) |  | 7 (8%) | 6 (6%) |  |
| Hospitalization between 31-90 days, n(%) | 1 (11%) | 0 (0%) |  | 5 (5%) | 8 (8%) |  |
|  |  |  |  |  |  |  |
| **If yes to Infection (N=44)** | **SPD**  **(N=3)** | **WPO**  **(N=0)** |  | **SPD**  **(N=21)** | **WPO**  **(N=20)** |  |
| Intravenous Antibiotic, n(%) | 3 (100%) | NA |  | 10 (48%) | 9 (45%) |  |
| Oral Antibiotic, n(%) | 2 (67%) | NA |  | 16 (76%) | 15 (75%) |  |
| Surgical Intervention, n(%) | 2 (67%) | NA |  | 14 (67%) | 14 (70%) |  |
| Close Observation only, n(%) | 0 (0%) | NA |  | 0 (0%) | 0 (0%) |  |

^*^P-value was corresponding to test the null hypothesis that P_WPO_ - P_SPD_  ≤ -0.06 for the infection related endpoints

where P_WPO_ and P_SPD_ are the true probability of infection for WPO and SPD group respectively. P-values for the remaining

analyses were not provided as the margin of inferiority was not pre-specified.
