# Supplementary material for: Antibiotics and Surgical Site Infection in Expander-Based Breast Reconstruction Trial (ASSERT)
Source: Ann Surg Oncol. 2025 Oct 14;33(4):3033–44. doi: 10.1245/s10434-025-18472-6 (PMC12982282; doi:10.1245/s10434-025-18472-6)
Supplement: Supplementary file 6 — Supplementary file6 (DOCX 15 KB) [file 10434_2025_18472_MOESM6_ESM.docx]

**Table Supplementary Digital Content 6: Antibiotics Used to Treat Infections**

| **Antibiotics used for treating Infection** | **SPD n=102** | **WPO n=112** |
| --- | --- | --- |
| trimethoprim/sulfamethoxazole, n (%) | 15 (14.7) | 11 (9.8) |
| ciprofloxacin, n (%) | 13 (12.7) | 12 (10.7) |
| vancomycin, n (%) | 11 (10.8) | 8 (7.1) |
| piperacillin/tazobactam, n (%) | 7 (6.9) | 4 (3.6) |
| cephalexin, n (%) | 5 (4.9) | 6 (5.4) |
| linezolid, n (%) | 5 (4.9) | 6 (5.4) |
| cefadroxil, n (%) | 4 (3.9) | 5 (4.5) |
| amoxicillin/clavulanate, n (%) | 3 (2.9) | 6 (5.4) |
| cefazolin, n (%) | 2 (2.0) | 1 (0.9) |
| clindamycin, n (%) | 2 (2.0) | 2 (1.8) |
| doxycycline, n (%) | 2 (2.0) | 6 (5.4) |
| levofloxacin, n (%) | 0 (0.0) | 1 (0.9) |
| daptomycin, n (%) | 0 (0.0) | 0 (0.0) |
| imipenem, n (%) | 0 (0.0) | 0 (0.0) |
| other, n (%) | 4 (3.9) | 6 (5.4) |
