# Supplementary material for: Antibiotics and Surgical Site Infection in Expander-Based Breast Reconstruction Trial (ASSERT)
Source: Ann Surg Oncol. 2025 Oct 14;33(4):3033–44. doi: 10.1245/s10434-025-18472-6 (PMC12982282; doi:10.1245/s10434-025-18472-6)
Supplement: Supplementary file 7 — Supplementary file7 (DOCX 15 KB) [file 10434_2025_18472_MOESM7_ESM.docx]

**Table Supplementary Digital Content 7: Association of Type of Skin Preparation and Type of Pocket Irrigation with SSI within 180 days**

| Parameter | Exp(B) | 95% Wald Confidence Interval for Exp(B) | | Sig. |
| --- | --- | --- | --- | --- |
|  |  | Lower | Upper |  |
| Type of skin prep (choice=chlorhexidine) | 0.750 | 0.243 | 2.319 | 0.617 |
| Type of skin prep (choice=chloraprep) | 1.667 | 0.747 | 3.719 | 0.212 |
| Type of skin prep (choice=betadine) | 0.958 | 0.407 | 2.258 | 0.922 |
| Type of skin prep (choice=other) | 0.958 | 0.407 | 2.258 | 0.922 |
| Type of pocket irrigation (choice=saline) | 0.726 | 0.333 | 1.583 | 0.421 |
| Type of pocket irrigation (choice=triple antibiotics) | 0.817 | 0.414 | 1.610 | 0.559 |
| Type of pocket irrigation (choice=bacitracin) | 1.289E-15 | 0.000 | 0.000 |  |
| Type of pocket irrigation (choice=betadine) | 0.920 | 0.374 | 2.265 | 0.856 |
| Type of pocket irrigation (choice=other) | 1.725 | 0.874 | 3.404 | 0.116 |
