# Supplementary material for: Antibiotics and Surgical Site Infection in Expander-Based Breast Reconstruction Trial (ASSERT)
Source: Ann Surg Oncol. 2025 Oct 14;33(4):3033–44. doi: 10.1245/s10434-025-18472-6 (PMC12982282; doi:10.1245/s10434-025-18472-6)
Supplement: Supplementary file 8 — Supplementary file8 (DOCX 14 KB) [file 10434_2025_18472_MOESM8_ESM.docx]

**Table Supplementary Digital Content 8: Association of Type of Drain Dressing with SSI within 180 days**

| Parameter | Exp(B) | 95% Wald Confidence Interval for Exp(B) | | Sig. |
| --- | --- | --- | --- | --- |
|  |  | Lower | Upper |  |
| Type of drain dressing (choice=Dry gauze) | 0.762 | 0.161 | 3.610 | 0.732 |
| Type of drain dressing (choice=CHG dressing) | 1.704 | 0.422 | 6.876 | 0.454 |
| Type of drain dressing (choice=Biopatch) | 0.559 | 0.232 | 1.345 | 0.194 |
| Type of drain dressing (choice=Xeroform) | 0.416 | 0.051 | 3.374 | 0.412 |
| Type of drain dressing (choice=NPWT) | 1 |  |  |  |
| Type of drain dressing (choice=Drain dressing antibiotic ointment) | 0.659 | 0.184 | 2.356 | 0.521 |
| Type of drain dressing (choice=Other) | 2.058 | 1.043 | 4.062 | 0.037 |
