# Supplementary material for: Antibiotics and Surgical Site Infection in Expander-Based Breast Reconstruction Trial (ASSERT)
Source: Ann Surg Oncol. 2025 Oct 14;33(4):3033–44. doi: 10.1245/s10434-025-18472-6 (PMC12982282; doi:10.1245/s10434-025-18472-6)
Supplement: Supplementary file 9 — Supplementary file9 (DOCX 16 KB) [file 10434_2025_18472_MOESM9_ESM.docx]

**Table Supplementary Digital Content 9: Association of Mastectomy Weight, TE Size, ADM Use, Position of Expander, Breast Cancer Status and Duration of Surgery with SSI within 180 days**

| Parameter | Exp(B) | 95% Wald Confidence Interval for Exp(B) | |  |
| --- | --- | --- | --- | --- |
|  |  | Lower | Upper | Sig. |
| Mean Mastectomy weight (g) | 1.001 | 1.000 | 1.002 | 0.033 |
| **SPD** Mean Mastectomy weight (g) | 1.000 | 0.998 | 1.002 | 0.948 |
| **WPO** Mean Mastectomy weight (g) | 1.002 | 1.001 | 1.004 | 0.005 |
| MeanTissue expander size (mL) | 1.002 | 1.000 | 1.005 | 0.105 |
| **SPD** MeanTissue expander size (mL) | 1.001 | 0.997 | 1.005 | 0.534 |
| **WPO** MeanTissue expander size (mL) | 1.003 | 1.000 | 1.006 | 0.067 |
| Acellular Dermal Matrix (ADM) used, Yes | 0.638 | 0.308 | 1.321 | 0.226 |
| **SPD** Acellular Dermal Matrix used, Yes | 0.966 | 0.335 | 2.785 | 0.949 |
| **WPO** Acellular Dermal Matrix used, Yes | 0.417 | 0.150 | 1.159 | 0.093 |
| Position of Expander_Prepectoral | 1.154 | 0.314 | 4.241 | 0.830 |
| **SPD** [Position of Expander_Prepectoral] | 0.592 | 0.136 | 2.570 | 0.484 |
| **WPO** [Position of Expander_Prepectoral] | 5.369E+09 | 0.000 | 0.000 |  |
| Breast Cancer Status_DCIS | 1.057 | 0.478 | 2.338 | 0.891 |
| Breast Cancer Status_Invasive | 1.321 | 0.660 | 2.645 | 0.432 |
| Duration of surgery (hours) | 0.961 | 0.766 | 1.205 | 0.730 |
